# Single Photon Emitters in Thin GaAsN Nanowire Tubes Grown on Si

**DOI:** 10.1021/acsnano.5c12139

**Published:** 2025-10-23

**Authors:** Nadine Denis, Akant Sagar Sharma, Didem Dede, Timur Nurmamytov, Salvatore Cianci, Francesca Santangeli, Marco Felici, Victor Boureau, Antonio Polimeni, Silvia Rubini, Anna Fontcuberta i Morral, Marta De Luca

**Affiliations:** † Physics Department, 243268 University of Basel, 4056 Basel, Switzerland; ‡ Physics Department, 9311 Sapienza Università di Roma, 00185 Rome, Italy; § Laboratory of Semiconductor Materials, Institute of Materials, 27218 EPFL, 1015 Lausanne, Switzerland; ∥ CNR - Istituto Officina dei Materiali (IOM), Laboratorio TASC, 34149 Trieste, Italy; ⊥ Physics Department, University of Trieste, 34127 Trieste, Italy; # Interdisciplinary Center for Electron Microscopy, 27218 EPFL, 1015 Lausanne, Switzerland; ∇ Faculty of Basic Sciences, Institute of Physics, 27218 EPFL, 1015 Lausanne, Switzerland

**Keywords:** semiconductor nanowires, single photon emitters, photoluminescence, GaAsN, dilute nitrides, time-resolved PL, second-order correlation function

## Abstract

III–V nanowire heterostructures can act as sources of single and entangled photons and are enabling technologies for on-chip applications in future quantum photonic devices. The peculiar geometry of nanowires allows to integrate lattice-mismatched components beyond the limits of planar epilayers and to create radially and axially confined quantum structures. Here, we report the plasma-assisted molecular beam epitaxy growth of thin GaAs/GaAsN/GaAs core–multishell nanowires monolithically integrated on Si (111) substrates, overcoming the challenges caused by the low solubility of N and a high lattice mismatch. The nanowires have a GaAsN shell of 10 nm containing 2.7% N, which reduces the GaAs bandgap drastically by 400 meV. They have a symmetric core–shell structure with sharp boundaries and a defect-free zincblende phase. The high structural quality reflects in their excellent optical properties. Local N% fluctuations and radial confinement give rise to quantum dot-like states in the thin GaAsN shell, which display remarkable single photon emission with a second-order autocorrelation function at zero time delay as low as 0.05 in continuous and in pulsed excitation.

## Graphical abstract

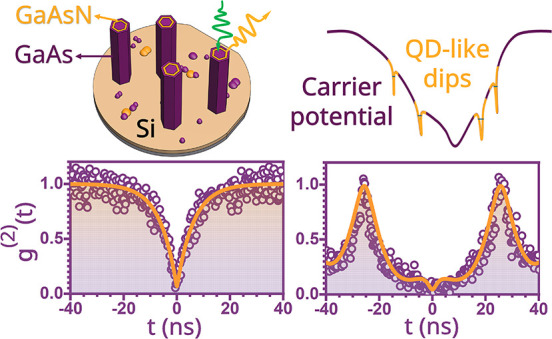

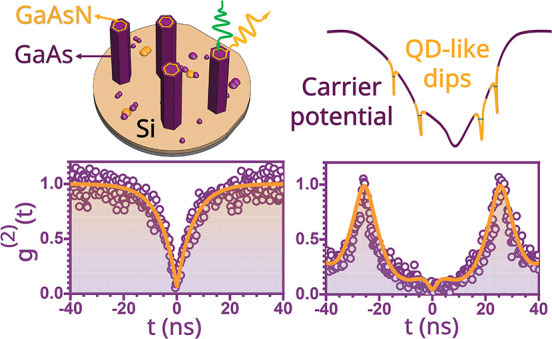

## Introduction

The peculiar growth, transport, and optical properties of semiconductor nanowires (NWs) have attracted significant research interest, making them valuable components for nanophotonic and quantum optical devices. NWs have emerged as a platform for applications, such as subwavelength lasers and ultrasensitive sensors,^1^ and may be integrated into quantum communication or quantum computation devices as sources of single photons^2–4^ and entangled photon pairs.^5^ Furthermore, NW-based devices can be integrated on-chip into an optical cavity or photonic crystal by growing them site-controlled or by individually moving them to a desired location.^4,6^

With NWs, in contrast to thin films, the superior physical properties of III–V materials — such as their high electron mobility and direct bandgap — can be achieved more easily by bottom-up growth onto standard Si substrates. This is due to the relaxed lattice mismatch requirements owing to a nm-sized interface between the NW and the Si surface.^7–9^ Moreover, NWs can accommodate strains in two dimensions due to the large surface-to-volume ratio, making them suitable for growing core–shell heterostructures with highly lattice-mismatched materials.^10–12^ During the NW growth, defects with long-range strain fields, such as vacancies and dislocations are typically attracted to nearby surfaces, making them inherently free of point and line defects,^13^ which affect carrier mobility and lifetime. Furthermore, in NW shells made of ternary alloys, such as the GaAsN-based heterostructures investigated here, strain can be compensated by radial variations in the alloy composition at the corners.^12,14–15^ For all these reasons, the NW geometry offers an enhanced ability to control the bandgap across radial and axial heterostructures while maintaining the high crystal quality required for nanoscale devices.

This work contributes to the search for a high-purity quantum-light source compatible with Si. Indeed, while self-assembled III–V quantum dots (QDs) provide high-performance single photon emitters (SPEs), it is difficult to grow them on Si with high quality and with position control. NWs, in contrast, can host site-controlled III–V SPEs grown on Si, but the SPE properties should be further optimized, and the range of available material systems further explored. This work introduces GaAs/GaAsN NWs to the panorama of III–V single photon emitters that can be monolithically integrated on Si.^16^ So far, this was achieved at wavelengths around 760 nm in AlGaAs/GaAs NWs,^17^ in the near-infrared around 900 nm in InAs/GaAs NWs,^18^ and at telecommunication wavelengths around 1340 nm in InAs/InP NWs.^19^ Here, we obtain SPEs with wavelengths around 950–1000 nm by using GaAs/GaAsN-based NWs. Using dilute nitride NWs as SPEs may also provide the basis for a future fine energy-control of the SPEs in NWs, exploiting the particular advantages of dilute nitrides.^20^ Specifically, here we achieve single photon emission from vertical NWs, which makes them especially suitable for vertical photonic devices.

Heterostructures made of a Ga­(In)­AsN alloy are used in particular for optical applications in the near-infrared range, as the GaAs bandgap decreases significantly when small amounts of N are incorporated. A dilute amount of N in GaAs creates a strong perturbation potential in the GaAs lattice, which leads to a splitting of the otherwise degenerate conduction band and drastically reduces the electronic bandgap. According to the band-anticrossing model (BAC),^21–23^ which is detailed in the SI II, the bandgap of GaAsN can be continuously reduced by 440 meV by varying the N concentration from 0 to 4%, see the calculated nitrogen-dependent bandgap energy in the inset of Figure 1c (orange line). At the same time, the electron effective mass increases,^24^ which reduces the spill-out of carriers in the shell and improves the thermal stability of the confinement. Interestingly, the bandgap reduction is reversible when the material is exposed to low-energy ionized hydrogen gas.^25^ This has been used in planar materials to tailor the electronic band structure and create site-controlled single photon emitters on GaAs substrates.^26,27^ In general, the growth of high quality dilute GaAsN is associated with considerable difficulties mostly due to the large miscibility gap of the constituents, related to the large lattice mismatch between GaAs and GaAsN, and the low solubility of N in GaAs. By lowering the growth temperature, thus growing GaAsN under nonequilibrium conditions, these problems may be partially overcome, leading to an increase in the N concentration.^28,29^ Nonetheless, by slightly increasing the growth temperature to an intermediate range, better optical qualities and less band-edge fluctuations are observed.^30^ Growing dilute nitride NWs with GaAsN in the core has not yet been achieved because the reduced growth temperature required by GaAsN leads to a reduced Ga mobility, which may prevent the Ga adatoms from diffusing from the NW sidewalls up to the Ga droplet at the tip.^31,32^ Furthermore, in the case of dilute GaPN, it has been argued that the N plasma increases the nucleation rate at the Ga-droplet/Si interface, resulting in planar growth instead of vertical NWs at the start.^33^ Despite all these difficulties, the growth of GaAs/GaAsN/GaAs with N concentration up to 3% has been reported in thick core–shell NWs,^34^ having a total diameter of 350 nm and a GaAsN shell thickness of 50 nm. Although these NWs exhibited twinning and random switching between wurtzite (WZ) and zincblende (ZB) phases, as well as phase separation for high N concentrations, they showed good optical emission properties at room temperature. Contrarily to GaAsN epilayers, incorporating N did not lead to an optical degradation compared to the GaAs reference NWs. Other studies explored the possibility of a patterned growth, for low N concentrations with 0.8% N and a diameter of 220 nm.^32^ Also, lately, NWs with a multiquantum-well GaAs/GaInAsN structure with optical emission at telecommunication wavelength were demonstrated, by alloying with indium.^35^ These growth efforts are motivated by the unique properties of dilute Ga­(In)­AsN, which have led to exceptional results for applications, such as lasing^36^ or spin filtering in N-rich nanopillars up to room temperature.^37^ In dilute GaNAsP NWs, it was possible to measure single photon emission, with second-order correlation function at zero delay (*g* ^(2)^(0)) values of 0.45 ^38^ at a wavelength of about 700 nm; however, single photon emission in GaAsN-based NWs has not yet been reported.

**Figure 1:**
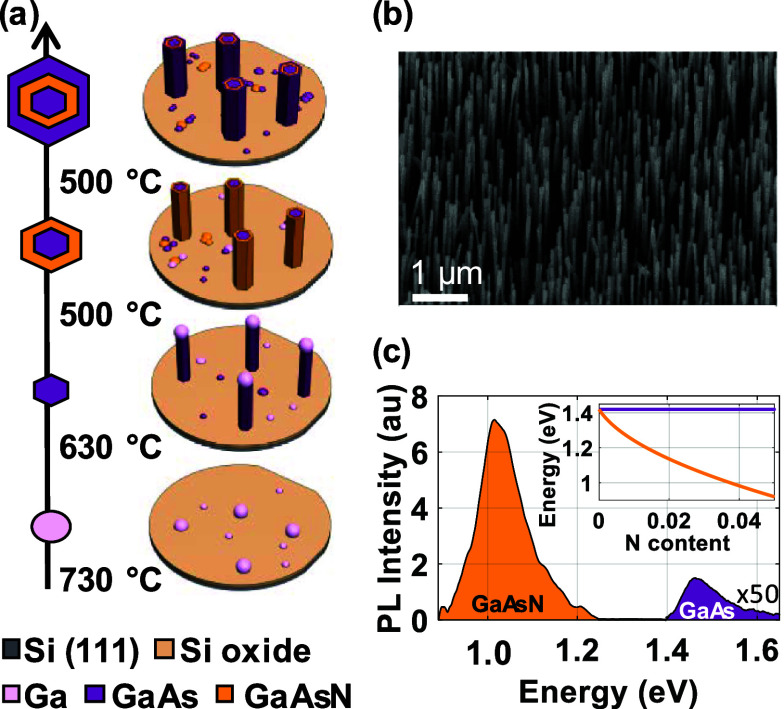
Growth and design of the GaAs/GaAsN/GaAs core–shell heterostructured NWs. (a) Illustrates the NW growth scheme: first, the nucleation of Ga droplets; second, the VLS growth of the thin GaAs core that is terminated with the controlled crystallization of the Ga droplet; third, the VS growth of the thin GaAsN shell at reduced temperature; and fourth, the VS growth of the GaAs outer layer. (b) SEM image of the NWs on sample A. (c) μ-PL spectrum at room temperature showing the bandgap emission of the GaAs core at 1.46 eV and the emission of the GaAsN shell at 1.02 eV. The inset shows the reduction of the GaAsN bandgap energy as a function of the N concentration according to the band-anticrossing model at room temperature for bulk (orange line), while the horizontal purple line marks the GaAs bandgap energy value as a reference.

In this work, we report the growth of thin GaAs/GaAsN/GaAs NWs on Si(111) substrates with a pure ZB phase along the NW axis and a nitrogen concentration of about 2.7%. The structural properties of the different NW samples were investigated by imaging with transmission electron microscopy (TEM). All the NWs have a pure defect-free ZB structure with a short WZ segment at the tip and a symmetric core–shell structure with sharp boundaries. The high crystalline quality of the NWs leads to μ-photoluminescence (PL) up to room temperature and to the emission of intense and narrow excitonic lines at low temperature. Finally, we achieve single photon emission by exploiting small variations in the N concentration and by designing the GaAsN active region very thin in order to favor quantum confinement of excitons. We measure a multiphoton emission probability below 6%, adding a new material system to the set of III–V pure single photon sources in NWs grown on Si.

## Results and Discussion

### Nanowire Samples

The designed NW heterostructure consists of a GaAs core with higher bandgap energy, surrounded by a thin GaAsN shell with lower bandgap energy and a second high bandgap GaAs outer shell. The NWs are grown by molecular beam epitaxy (MBE) on a Si(111) substrate. A schematic of the NW growth process and resulting geometry is shown in Figure 1a. First, the GaAs core is grown via a Ga-assisted vapor–liquid–solid (VLS) approach at 630 °C. Second, the substrate temperature is reduced to 500 °C for the epitaxial vapor–solid (VS) growth of the GaAsN and GaAs shells. We have grown two different samples: sample A has a GaAs nominal core diameter of 20 nm, a GaAsN shell of 10 nm, and a GaAs outer shell of 10 nm. Sample B has a thicker core of 40 nm and the same shell thicknesses as sample A. The scanning electron microscopy (SEM) image of the NWs from sample A in Figure 1b shows that the NWs are uniform, straight, and vertical, with an average length of approximately 2 μm. The μ-PL spectrum of the NW ensemble at room temperature in Figure 1c reflects the designed heterostructure. The bandgap of the GaAsN shell is decreased by 400 meV with respect to the emission energy of pure ZB GaAs at 1.42 eV, down to 1.02 eV. The thin GaAs core emits at 1.46 eV. The full width at half-maximum (fwhm) of the GaAsN emission is relatively large, with 100 meV compared to typical values in the range of 50 meV in optimized GaAsN epilayers. The broadening might be due to some N concentration or strain fluctuations in the radial direction of the NWs, these are typical in the corners of ternary alloy NW shells.^12,14^ According to the BAC model for bulk, we find a N concentration of 3.4%. However, the true N concentration is expected to be lower, because the smaller lattice constant of GaAsN with respect to GaAs causes a tensile strain in the shell that further reduces the bandgap. This is mirrored in the upshift of the GaAs emission energy by 40 meV due to a corresponding compressive strain in the core. Such strain distributions via elastic deformation of both, the shell and the core, are typical for thin core–shell NW heterostructures with a large lattice mismatch and they have a big impact on the bandgap.^7,12^ For a reduction of the bandgap due to strain in a range of 30–70 meV and considering a blueshift of approximately 25 meV due to quantum confinement in a thin GaAsN quantum well^22^ the N concentration in the shell is between 2.8 and 3.4%.

### Structural Characterization

First, we analyzed the structure of single NWs from Samples A and B by transmission electron microscopy (TEM). Figure 2 shows a representative structural characterization of a NW from sample A. Panel (a) shows the bright-field (BF) TEM image of the NW observed in ⟨110⟩ zone axis together with the selected area electron diffraction (SAED) pattern, (b)–(d) show the high resolution (HR) TEM images from the central region of the NW and in (e)–(g) show the HR-TEM images from the tip region. HR images from the bottom part of the NW and further characterizations of NWs from sample B are presented in the Supporting Information (SI1–SI4). The NWs from sample A and B show a pure and defect-free ZB section and only the bottom and the tip contain twin planes and stacking faults. These are common at the bottom of self-catalyzed NWs as a result of the contact angle instability of the initial droplet.^39,40^ It is possible to minimize these by growing at higher temperatures and lower Ga fluxes.^41^ The short WZ segment at the tip is created during the droplet consumption under excess As flux when the contact angle changes. At contact angles between 100 and 125 degrees, the WZ phase is favorable, whereas, at smaller and larger contact angles, the ZB phase is favorable.^42,43^ The high crystal purity and phase stability in the central region are achieved by keeping the diameter of the NW small, such that the droplet is stable over a wide V/III ratio.^44^ For thicker core diameters ZB and WZ have a tendency to coexist along the growth direction of the NW.^45,46^ The surrounding GaAsN and GaAs outer shells inherit the crystal structure of the core for these small diameters and N concentrations. We do not observe tapering in the NW diameter along the growth axis. Sample B has similar structural characteristics as sample A.

**Figure 2:**
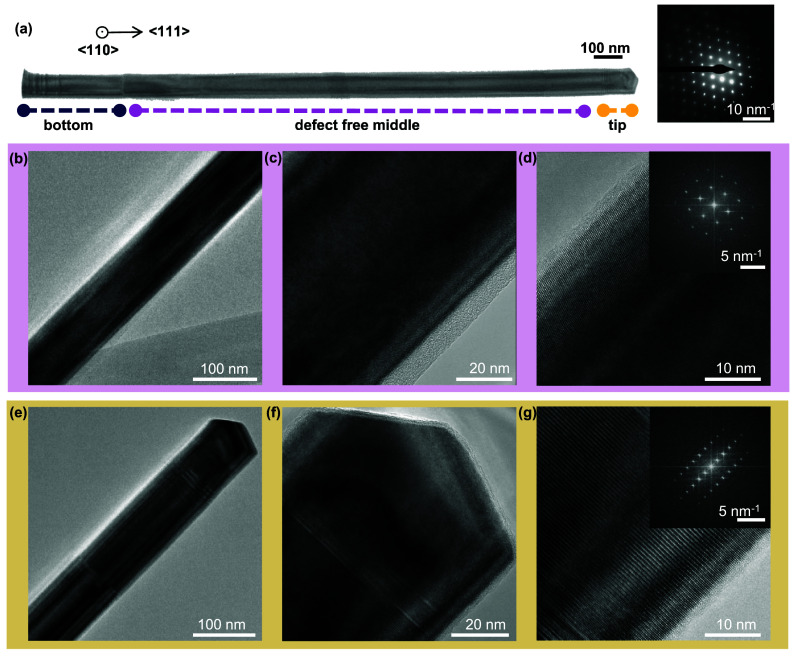
Structural analysis of a single NW by TEM. (a) BF-TEM image of an entire NW with an inset of the SAED pattern. (b–d) HR-TEM images from the middle region with a pure defect-free ZB phase and (e–g) HR-TEM images from the tip of the NW with a short WZ segment. The insets in (d) and (g) display the fast Fourier transform showing the ZB phase in (d) and the WZ phase in (g). These images are taken from the ⟨110⟩ zone axis on a NW transferred from sample A.

Next, we investigated the core–shell structure by imaging the axial cross-sectional cuts of the sample prepared by ultramicrotomy. Figure 3a,b show atomic-resolution images of the sample by annular dark-field (ADF) scanning transmission electron microscopy (STEM). The GaAs/GaAsN/GaAs core–multishell geometry has a well-defined hexagonal shape with {110} sidewalls. The GaAs core thickness of NWs from samples A and B is 23 and 42 nm, respectively (SI5 shows the precise measurements). The GaAsN shell thickness is ∼10 nm in both samples, which is very close to their nominally defined size. A distinct brighter contrast is observed along three out of six 112 planes laying in the symmetry axes linking the corners of the hexagonal shape of the NW. These may be caused by polarity-driven segregation on the ⟨112⟩ directions, which can be either A or B polar, depending on whether they are terminated by group III or group V compounds. Similar phenomena have been observed in various ternary alloy core–shell structures, where variations in the material composition could be measured,^12,14,47^ see SI6 and SI7 for further measurements and discussions. Finally, the compositions within the NW heterostructure is analyzed with energy dispersive X-ray spectroscopy (EDX). Quantitative elemental maps of the NW cross-section are shown in Figure 3c. For the GaAsN shell, the mean values of Ga, As, and N concentrations are 50.7, 46.4, and 2.9 atomic % respectively (see also EDX in SI8). This N concentration in the shell is in good agreement with the PL data in Figure 1 and matches the strain analysis results obtained by geometric phase analysis (GPA), which estimates approximately 2.5% of N, see details in SI8 and SI9.

**Figure 3:**
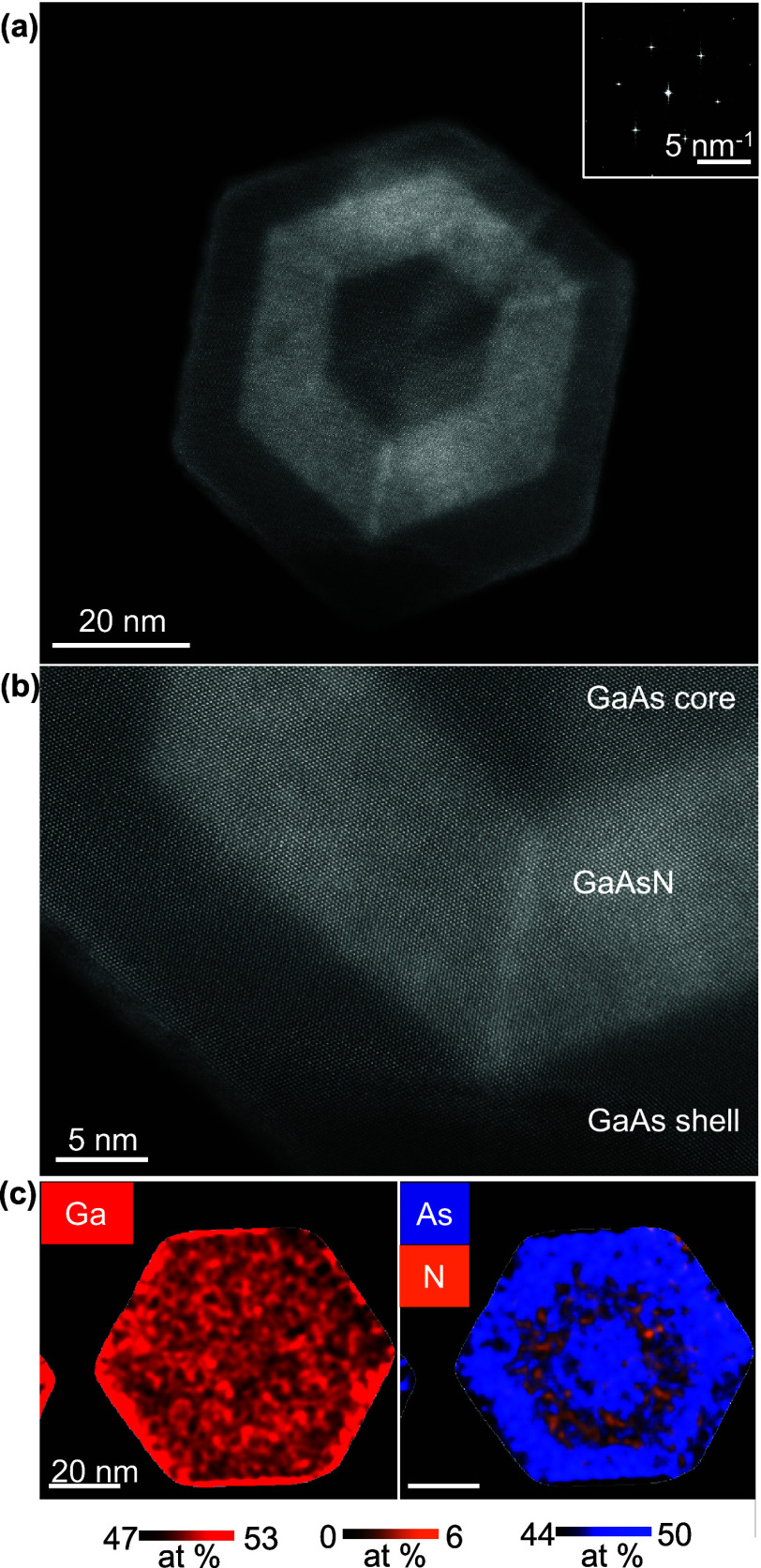
Structural and compositional analysis of the multishell cross-section of sample A. (a,b) Atomic-resolution ADF-STEM images from the ⟨111⟩ zone axis. The Fourier transform in the inset of (a) evidence the ZB crystal structure. A brighter contrast is observed in the GaAsN shell along three out of the six 112 planes laying in the symmetry axes linking the corners of the hexagonal section. (c) EDX maps of the atomic concentrations of Ga, and As and N, measuring a N concentration of 2.9% in the shell.

### Optical Properties of GaAsN Excitons

First, the optical properties of the NWs are investigated directly on the NW ensemble on the Si growth chip. Figure 4a shows the μ-PL spectrum at 6 K from the very thin NWs of sample B with a GaAsN shell thickness of 10 nm. The spectrum has two emission bands, the low-energy one from the GaAsN shell and the high-energy one from the GaAs core, which had to be multiplied by the factor provided in the figure in order to become visible. The spectral position of the GaAsN shell emission at 1.09 eV remains constant across different points on the sample, suggesting a uniform N composition across different NWs (see SI12 in SI section III for further spectra from samples A and B). The GaAs core emission is more point-dependent and centered between 1.53 and 1.57 eV for sample A, with a thinner core diameter of 20 nm, and 1.51–1.53 eV for sample B, with a core diameter of 40 nm. These are relatively high energies compared to the GaAs bulk of 1.515 eV or GaAs NWs with short politypic regions with emission bands between 1.51 and 1.52 eV.^48^ As discussed in SI12, the shift to higher energy for thinner GaAs core diameters may be affected by two mechanisms, one related to a high thermalization temperature of charge carriers, previously observed in GaAs NWs,^49^ and the other one related to the presence of compressive strain in the core, due to the lattice mismatch between the constituents.^7,12^ Emission peaks around 1.57 eV have been measured before on the polytypic tips of GaAs NWs.^50^

**Figure 4:**
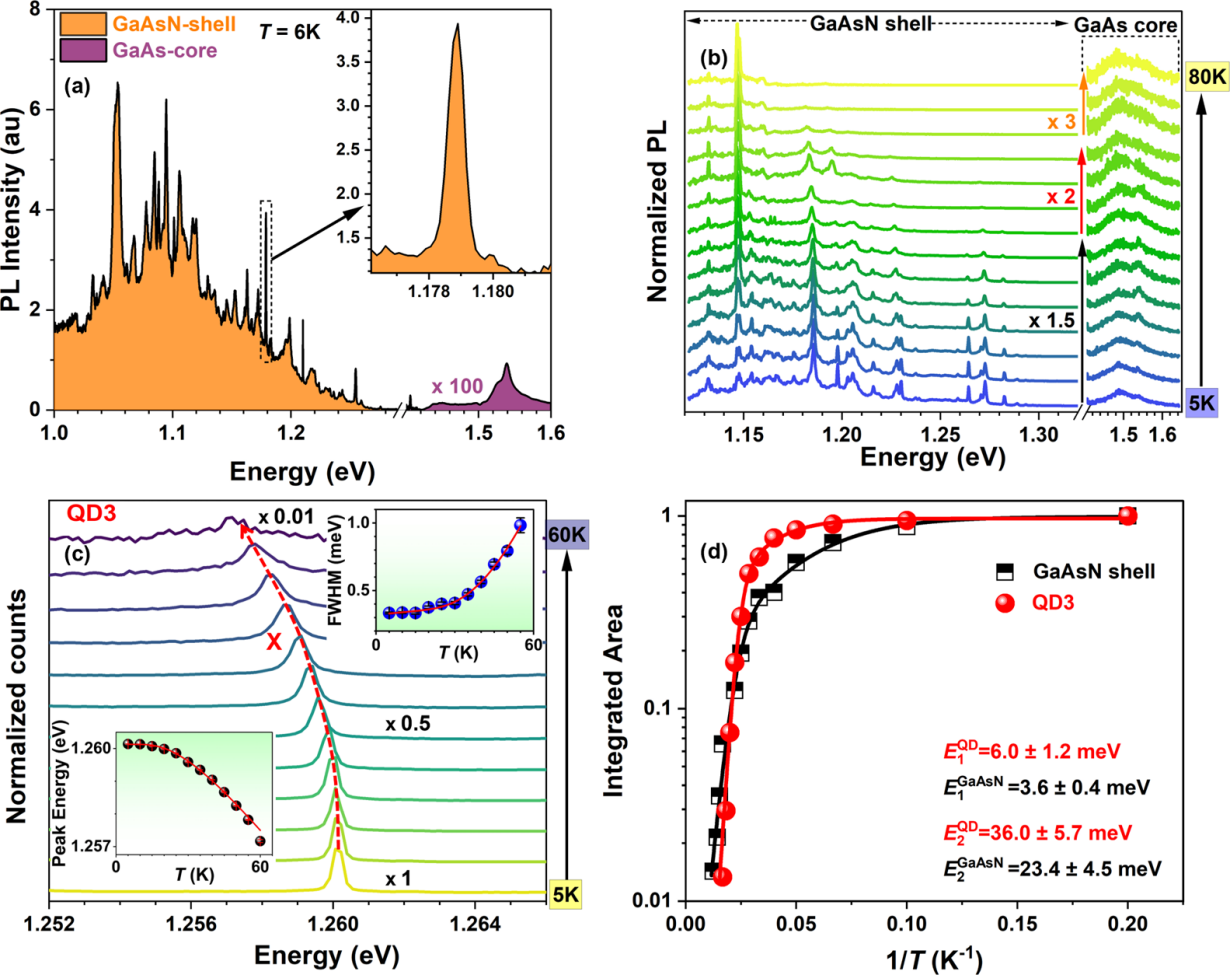
Temperature-dependent optical properties of the GaAs/GaAsN/GaAs NWs. (a) μ-PL spectrum from the growth chip of sample B at *T* = 6 K and 4 μW power (power density 1600 W/cm^2^). The emission from the 10 nm thick GaAsN shell is colored orange, while the emission from the 40 nm thick GaAs core is colored purple and multiplied by a factor of 100 for better visibility. The inset presents a zoomed plot of a typical QD-like emission, peaked at 1.179 eV. (b) Temperature-dependent normalized PL spectra taken on a different point on sample B, conducted at the power of 1 μW, showing GaAsN and GaAs emission bands, as indicated. The GaAs emission energy above 1.4 eV is multiplied by a factor 100 and by the given factors, depending on the temperature, for improving visibility. The arrow indicates the temperature increase from 5 to 80 K with a step of 5 K up to 60 K and a step of 10 K after 60 K. (c) Temperature-dependent normalized PL spectra of a single isolated QD emission line (X exciton) centered at 1.260 eV (QD3) (distinct from the QD line in the inset in panel (a)), measured with 5 K steps on sample B at 1 μW excitation power. Luminescence intensity (800 counts/s at 5 K) decreases by about 2 orders of magnitude with increasing temperature, as shown by normalization factors given in black for three representative spectra. The insets illustrate the evolution of QD emission energy (bottom left) and fwhm (top right) with temperature, both with the respective overimposed fitting curve. (d) Arrhenius plot comparing the thermal quenching of QD3 emission with the GaAsN shell emission (band illustrated in panel (b)), both fitted with a two-activation energy model, whose resulting energy values are given.

The GaAsN shell emission dominates the spectrum in all points of samples A and B, with a PL intensity up to 3 orders of magnitude higher than that of the GaAs core. This is due to an efficient diffusion of excitons from the high-energy GaAs core to the low-energy GaAsN shell,^51,52^ considering that typical exciton diffusion lengths are larger than the NW radial dimensions. The temperature study of a typical point on sample B is shown in Figure 4b, where both the GaAsN and the GaAs bands are visible from 5 to 80 K. The total PL emission decreases by more than an order of magnitude when heating the sample to a temperature of 80 K. The weight of the high-energy side of the GaAsN emission is progressively decreased as temperature increases, as trapped carriers are thermally activated into the delocalized states, where they move to lower energy states or nonradiative recombination centers. The pronounced sharp peaks on the low-energy emission band are typical for the GaAsN material. They are created by short-range N-concentration fluctuations, leading to three-dimensional confinement of the carriers in QD-like states, as observed in bulk GaAsN^53^ with N concentration of 3% and in thick GaAs/GaAsN NWs with low N concentration (0.5%),^51,54,55^ for which, however, no single photon emission has been measured. This type of isolated emitters arising from N fluctuations are, indeed, typically referred to as “QD-like emitters”. In bulk, single photon emission in GaAsN has been observed from luminescent centers that are localized on impurities or N-complexes for very dilute N concentrations up to 0.3%.^56–58^ Typically, they emit at well-defined emission energies, ranging between 1.48 and 1.51 eV. However, the highly localized states in our NW samples have instead different emission energies, and they are distributed over the GaAsN band due to random N concentration fluctuations. In Figure SI11, in section II of the SI, we illustrate the band diagram corresponding to QD-like potential dips in the GaAsN shell created by local fluctuation of N concentration. The QDs in these samples have a typical PL line width between 250 and 700 μeV. The temperature study shows that most of the excitons are trapped up to a temperature of 50 K when most of the narrow lines disappear, with only low-energy lines remaining. Figure 4c illustrates the evolution of the PL spectrum of a typical QD-like emitter as a function of temperature. For this study, and for the following single photon emission studies, we focus on emitters located on the high-energy side within the GaAsN band, because they are spectrally isolated, not being influenced by the broad GaAsN bandgap emission nor by emitters at similar energies. At 5 K, this QD exhibits an emission energy of 1.261 eV (QD3), with a fwhm of about 250 μeV. The QD emission energy experiences a redshift with increasing temperature, accompanied by broadening, while simultaneously losing PL intensity. A similar temperature-dependent investigation was carried out, for statistical purposes, on two additional single isolated QDs with emission energy 1.275 eV (QD1) and 1.257 eV (QD2), and the results are presented in Figure SI13 in the SI (section IV). The PL signal from all the QD emissions is quenched at a temperature around 50–70 K. The redshift and the fwhm broadening of the QD emission with temperature are presented in the inset of Figure 4c. The peak shift with temperature has been fitted with the one-oscillator model,^59^ discussed in SI IV. The average phonon energy obtained from this model for QD1 is 7.3 meV, and ranges between 6 and 10 meV for all the QDs (see Figure SI14). The fwhm shows typical QD behavior:^60^ at low temperatures (5–15 K) it remains fairly constant, as the temperature increases it starts to broaden due to increased exciton–phonon interaction and carrier scattering. To extract the effective strength of the exciton–phonon coupling contributing to the line width broadening, we have employed the exciton–phonon coupling model^61^ to fit the temperature-dependent fwhm (see Figure SI14 and SI section IV). From the fitting, the exciton-acoustical phonon and the exciton-optical phonon coupling constants were obtained for all the QD lines, with values comparable to those of other QDs.^60,62,63^

The Arrhenius plot in Figure 4d illustrates the integrated intensity of the GaAsN emission band (shown in (b)) and of the QD3, both fitted with a common two-activation energy model.^64^ The derived activation energies *E*_1_ and *E*_2_ for all the QDs range between 3 and 6 meV and 30–40 meV, respectively (Figure SI14). The value for *E*_1_ is consistent across all the QDs and the GaAsN shell emission, the *E*_2_ value is instead higher in QDs than in the GaAsN shell emission, which suggests a higher carrier trapping in the potential dips. The details of the fitting equations and further discussion are provided in the SI IV.

To gain further insight into the optical properties and the nature of these emitters, the spectral features of the QDs were monitored for increasing values of the excitation power, as summarized in Figure 5a. This figure is relative to QD1, whose temperature study can be found in Figures SI13 and SI14. In QD1 (as well as in other QDs, whose power studies are in Figure SI15 in the SI, section V), the increasing power leads to a slight redshift, due to laser-induced heating and to a Varshni-like shift of the bandgap, and to line width broadening, possibly related to spectral diffusion (as further discussed in SI18). The high-power spectra in Figure 5a also show a low-energy peak, XX, further analyzed in Figure 5b, which shows the integrated area of the investigated line for increasing excitation levels, featuring a saturation behavior typical of quantum emitters and accurately described by the fitting function *I* = *I*_sat_[*P*/(*P* + *P*_N_)] (where *I*_sat_ is the saturation intensity, *P* is the laser power and *P*_N_ is the laser power for which the intensity is half of the saturation value).

**Figure 5:**
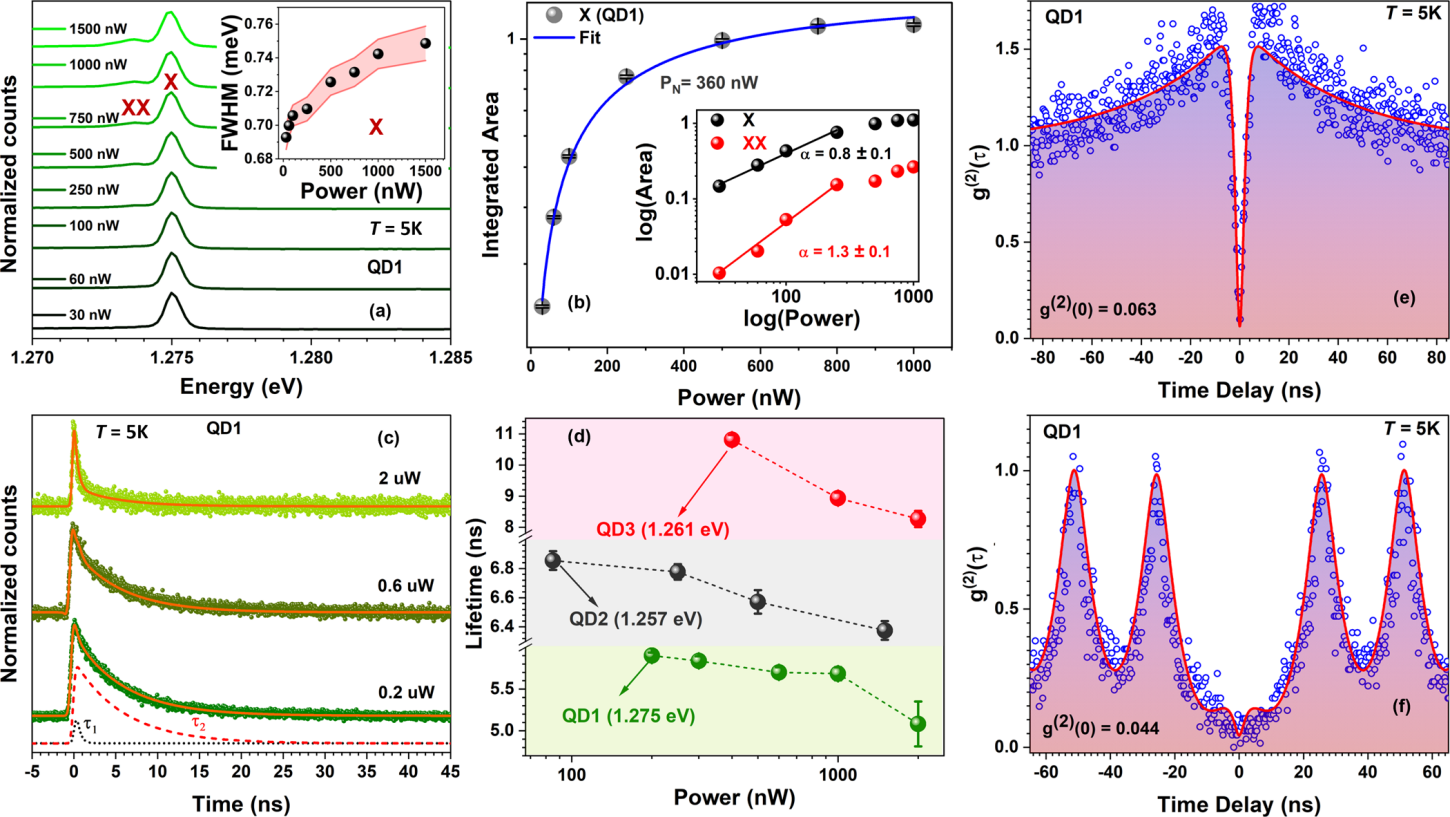
Low temperature optical properties and single photon emission of a QD in the GaAsN shell. (a) 5 K power-dependent study of a typical sharp excitonic QD-like emission line (QD1) peaked at 1.275 eV, measured on sample B. At high excitation powers, the emission spectra can be resolved into two excitonic features: exciton (X) at 1.2750 eV and biexciton (XX) emission at 1.2735 eV. At 30 nW, the intensity value of the X line is 200 counts/s and at 1000 nW, 1400 counts/s; the counts of the other spectra can be derived from panel (b). The inset shows the X line width broadening with power; the shadowed region indicates the error bars extracted from fitting. (b) Integrated area of the X peak as a function of power, saturating at high powers like a typical two-level system. The inset shows, in a log–log scale, a linear increase in the X and XX integrated areas at low power, with a slope for the XX peak nearly double compared to the X line. (c) μ-TRPL decay traces measured as a function of power at 5 K, fitted with a biexponential model, resulting in the orange line. The dashed lines (in the lowest power data) indicate the individual transient processes’ fast decay (τ_1_ = 0.38 ns) and slow decay (τ_2_ = 5.90 ns); the two curves are offset for clarity. (d) Decay time of the slower component for three different QDs as a function of power. (e) Second-order CW autocorrelation measurement and fit of QD1 at 5 K and power 0.5 μW. Single-photon emission is confirmed by a value of *g* ^(2)^(0) = 0.063. (f) Second-order autocorrelation function measurement and fit under pulsed laser excitation for the same QD, close to saturation power (0.6 μW). The amplitude of the central peak is 0.04, confirming single photon emission.

The inset displays the integrated areas of the two peaks visible in panel (a), on a log–log scale. Clearly, at low excitation power, the peaks have a linear behavior, with slopes of 0.8 and 1.3. The peak with the smaller slope is identified as a neutral exciton (X), while the low-energy peak with an almost double slope is identified with a biexciton, XX.^27^ Similar analysis for some additional QDs is presented in Figures SI16 and SI17. Figure 5c shows time-resolved μ-PL (μ-TRPL) decay traces of the X line of QD1. The measurements were performed as a function of power, at five power values from low (0.2 μW) to complete saturation power (2 μW), to gain further insights into the carrier confinement properties. A similar analysis was also performed on several other QDs, see Figure SI19 in the SI (section VI). In Figure 5c we present decay traces at three power values, with the corresponding fit taking into account the transient behavior of the exciton. This transient nature is well fitted by a biexponential decay function. The equation is discussed in section VI of the SI and the result is the orange curve, arising from the two dashed lines corresponding to two decay times, with the first decay lifetime (τ_1_) ranging from 0.3 to 0.4 ns and the slower decay lifetime (τ_2_) ranging from 5 to 11 ns in the different QDs. The faster decay, likely related to nonradiative processes, cannot be resolved very well by our setup (indeed, τ_1_ corresponds to the instrument response time), while the slower decay time agrees reasonably well with the previously reported exciton lifetime for III–V-nitride QDs in similar NWs.^65^ The exciton lifetime plotted in Figure 5d for three different QDs slightly decreases with increasing power. Such decrease suggests that at higher powers the excitonic ground state is fully occupied, leading to an increase in the overlap of the electron–hole wave functions and thereby a slight decrease in the radiative lifetime.^66^

Before moving to the demonstration of highly pure single photon emission from our QDs, we discuss below possible reasons for their lifetime values. An exciton lifetime of the order of 6 ns is long if compared to the decay times of QD-based single photon sources in NWs, micropillars or photonic cavities, ranging between 0.08 and 2.4 ns,^67^ and also to the lifetime of typical self-assembled GaAs/AlAs or InGaAs/GaAs QDs.^68,69^ Slow exponential decay times of localized excitons in GaAsN have been observed before, with a characteristic time of 10 ns in epilayers^70^ and 5.2 ns in NWs.^71^ QDs in GaAsN epilayers that are engineered by site-selective hydrogenation have shorter decay times, equal to 1–3 ns.^27^ A range of decay times has also been observed in excitons bound to N-complexes in GaAs, from approximately 0.8 ns^72^ to more than 10 ns.^58^

For III–V-nitrides NWs the exciton lifetime can be influenced by several factors, some of which are discussed in ref 65. One factor could be the strong localization in the potential dips of the conduction band, which reduces the probability of trapping by fast nonradiative centers. However, the observed long decay time may also indicate that the e-h pairs are spatially separated and that their radiative recombination rate is, therefore, low due to the weak overlap of their wave functions. Such separation could arise because the electrons are trapped in the N-induced potential dips and the holes are free to move, or because also the holes are trapped in band potential dips, such as those caused by WZ inclusions or other defects, which occur away from the electron potential dips.

Another possible reason for the long lifetime is that the strain and local anisotropy experienced by the QD in the GaAsN shell may favor light-hole states, reducing the oscillator strength of the emitters and also resulting in a slow recombination lifetime. It is also worth noticing that the diameter of these thin NWs does not support waveguide modes that typically accelerate the emission rate, thus decreasing the lifetime.

Finally, we clarify that, despite the optical measurements being performed on vertical NW ensembles on their growth chip, we can rule out that our signal arises from the substrate. Indeed, cathodoluminescence measurements (shown in Figure SI20 in the SI VII) clearly confirm that the emission from both the GaAsN shell and the GaAs core is from single vertical NWs connected to their growth substrate and not from interstitial layers or substrate. As further discussed in the SI (section VII), the single thin NWs from sample A and B did not show PL emission when lying horizontally, due to heat dissipation issues typical of thin GaAs-like NWs.^73,74^

### Single Photon Emission from Quantum Dots in the GaAsN NW Shell

To prove that the localization of carriers in these highly confined structures results in the emission of pure single photons, we measured the second-order autocorrelation function *g* ^(2)^(τ) of one of the spectrally isolated lines on the high-energy side of the GaAsN emission band of sample B. The *g* ^(2)^(τ) was measured with a Hanbury Brown and Twiss setup under a continuous-wave (CW) excitation on QD1 at *P* = 0.5 μW, which is below saturation of the QD.^2^ Figure 5e shows the normalized coincidence counts as a function of the time delay. A pronounced antibunching at zero time delay is visible, with a value of *g* ^(2)^(0) = 0.063, well below the threshold of 0.5, for which a state emits only one photon per cycle of excitation. The antibunching dip at *g* ^(2)^(0) returns to the asymptotic value of 1 for time delays of several ns, which agrees well with the relatively slow recombination dynamics investigated with time-resolved PL measurements. For statistical reasons, in the SI section VIII we show *g* ^(2)^(τ) measurements on two additional QDs, displaying antibunching, as well as the procedure required to remove setup crosstalk (Figure SI21).

As further explained in the SI section IX, the fit that is overimposed to the data in Figure 5e not only allowed determining the *g* ^(2)^(0) value, but it also enabled us to investigate the time evolution of the carrier population inside our QDs (e.g., the carrier capture time), as it arises from the solution of a system of rate equations.^26^ To reproduce the broad bunching peaks visible on both sides of the antibunching dip at τ = 0, an additional metastable state was introduced in the equations (see details in SI, section IX).

On the same QD, we also performed *g* ^(2)^(τ) measurements under pulsed laser excitation, shown in Figure 5f. Remarkably, at τ = 0, the function is 0.044, further highlighting the pure nature of these single photon emitters. The overimposed curve was obtained, as explained in SI IX, by solving a nearly identical system of rate equations. However, the curve is not a fit, as there are no free fitting parameters, we only used as parameters the values obtained from fitting the time-resolved and CW autocorrelation data. Despite the absence of free fitting parameters, this curve overlaps well with the experimental data, further corroborating our analysis.

The small values of *g* ^(2)^(0) both in CW and in pulsed excitation demonstrate a high purity for single photon emission in a system without any performance-enhancing cavity and underline the high quality of the material. Future growth processes involving the optimization of NW diameter and tapering angle could promote cavity or waveguiding effects and decrease lifetime while also increasing QD efficiency. The efficiency of the current QDs is about 1%, as discussed in the SI section X. These GaAsN-based NW QDs, whose energy can potentially be finely tuned by postgrowth hydrogenation—as was achieved in planar quantum wells^20,26,27^ —are particularly promising for future postgrowth integration in external spectrally matched narrow-band cavities. It is also worth noticing that this new material system can provide, in the near future, telecom SPEs, by alloying with In as it was done in thick NWs.^35^

In conclusion, the observation of single photon emission in these NWs (a first for GaAsN-based wires, as we have noted) can be attributed to two of their defining characteristics: the reduced thickness of their GaAsN shell, which further promotes confinement to carriers localized by the dips in the potential landscape caused by N concentration fluctuations, and their high crystalline purity, characterized by extended ZB defect-free regions, which had not been achieved before in GaAsN NWs. The thin shell itself contributes to the high crystal quality, as it minimizes the impact of lattice mismatch and strain typical of these materials.

## Conclusions

In this work we have demonstrated the growth of thin GaAs/GaAsN/GaAs core multishell NWs via plasma-assisted MBE on Si, which makes the material system suitable for future Si-based quantum photonic circuit applications. The structural properties were analyzed by atomic resolution TEM and ultramicrotomy sectioning of the NWs. We achieved a high crystalline quality with a defect-free ZB phase along the main part of the NW and a short WZ segment at the top of each NW. The low temperature μ-PL spectra show very narrow excitonic lines from quantum dot-like states. This study shows the importance of the low thickness of the GaAsN shell in order to create pure single photon emitters. For the first time, we measure quantum-light emission from GaAsN-based NWs, obtaining a *g* ^(2)^(τ) value at zero time delay of 0.06 in CW and 0.04 in pulsed excitation. In conclusion, we have designed a NW system that can be suitable to create photonic devices based on single photon emitters monolithically integrated on Si.

## Experimental Section

### Growth

The NW samples were grown by molecular beam epitaxy (MBE) in a Riber ^32^P system equipped with Ga and As effusion cells and a radio frequency (RF) plasma source fed by a mixture of ultra pure N_2_ and Ar gases. Si (111) As-doped wafers were utilized for all samples. Samples A and B were grown on the thin native oxide layer. NWs were grown by combining Ga-assisted VLS growth for the GaAs core and VS epitaxial growth for the GaAsN and GaAs shells. The overview of the growth procedure is depicted in Figure 1a. In situ surface modification procedure (SMP)^75^ was employed for samples A and B to ensure the formation of homogeneous Ga nanoparticles (NPs) assisting the NW core growth. The procedure was as follows: first annealing at 730 °C for 30 min, deposition of 3 monolayers (MLs) of Ga at 600 °C, and second annealing at 730 °C for 5 min. At the end of NW growth, Ga droplets were crystallized in GaAs under As flux.^76^ The substrate temperature was then lowered to 500 °C for GaAsN and GaAs shells for all samples. The growth details of NW samples are summarized in SI14. XRD measurements of pseudomorphic thin film samples deposited under the same conditions as these NWs provide a nominal N concentration of 1.5% for sample A and 0.9% for sample B. However, the effective N incorporation may differ,^77^ as discussed in the SI23.

### Imaging

NWs were transferred onto a copper grid and analyzed using Thermo Fisher Scientific TEMs, Tecnai Osiris and Talos F200S, operated at 200 kV. Ultramicrotome cuts are created by the Leica EM UC7 Ultramicrotome system. The structures were embedded in epoxy resin and peeled out from the substrate. A Diatome ultra 35° diamond knife was used to obtain smooth cross sections, with the thickness of each cross-section aimed to be 80 nm. Atomic-resolution images of the NWs cross-section were obtained using an aberration-corrected FEI Titan Themis STEM operated at 300 kV; more information about the EDX and GPA methods can be found in the SI.

### Optical Measurements

The μ-PL measurements were performed using a 532 nm solid-state laser (DPSS) with controlled excitation power to limit the heating and damage of the NWs. The light was focused through a 100x objective with NA = 0.75, resulting in a diffraction-limited spot size of 750 nm. The signal was collected in the backscattering geometry through a 0.75 NA microscope objective, dispersed by a 0.5m long spectrometer, and detected by a liquid nitrogen cooled CCD and InGaAs detector. All measurements were normalized for the spectral response of the setup collected using a blackbody light source with known spectrum. The pumping power is 10 μW for room temperature measurements and below 4 μW for measurements taken at 5–6K. The spot size captures emission from approximately 4–8 NWs. For cryogenic measurements, samples were placed either in a continuous-flow He cryostat or in a closed-cycle He cryostat at a temperature of 5–6 K, and moved using a piezoelectric stage with 100 nm precision.

For the *g* ^(2)^(τ) measurements, the exit slit of the spectrometer was set to limit the spectral bandwidth to 1.5 nm, centered on the quantum emitter line. For measuring the coincidence counts, the dispersed signal was collimated by a parabolic mirror into a Hanbury Brown and Twiss setup. A 50/50 beam splitter divided the light into two branches that were collected by two Si avalanche photodiodes (APDs) that were interfaced with a PicoHarp 300 time-correlated single photon counting (TCSPC) module (maximum resolution of 4 ps). The time resolution of the setup is about 500 ps, primarily limited by the combined time resolution of the APDs. Cross-talk-induced bunching peaks were eliminated by comparing them to the coincidence counts of the μ-PL emitted from an InP sample without single photon behavior, as shown in the SI21.

For the time-resolved μ-PL measurement, we used a supercontinuum pulsed laser with a 50 ps pulse width and a repetition rate of 39 MHz, tuned at 520 or 525 nm with acoustic-optic tunable filters. A beam sampler on the excitation path provided the START signal for measuring the time-difference at a photodiode interfaced with the TCSPC module, and the μ-PL signal from the sample is focused onto a single avalanche photodiode, providing the STOP signal. The decay curve of the emitter yields a lifetime which is considerably longer than the instrument response function (420 ps for our setup), so no deconvolution procedure was performed.

## Supplementary Material



## Notes

Structural characterization of samples A and B; HR-TEM of nanowire tips and of bottom parts; EDX of sample A; strain analysis; cathodoluminescence; additional photoluminescence measurements as a function of power, temperature, and time-resolved; *g* ^(2)^(τ) measurements and modeling; and QD estimation efficiency (PDF)
