# Supplementary material for: Single Photon Emitters in Thin GaAsN Nanowire Tubes Grown on Si
Source: ACS Nano. 2025 Oct 23;19(46):39757–67. doi: 10.1021/acsnano.5c12139 (PMC12659438; doi:10.1021/acsnano.5c12139)
Supplement: Supplementary file 1 [file nn5c12139_si_001.pdf]

Supporting Information

Single photon emitters in thin GaAsN nanowire tubes grown on Si

Nadine Denis,[†] *Akant Sagar Sharma,*[‡] *Didem Dede,*[¶] *Timur Nurmamytov,*[§,∥]
Salvatore Cianci,[‡] *Francesca Santangeli,*[‡] *Marco Felici,*[‡] *Victor Boureau,*[⊥] *Antonio Polimeni,*[‡] *Silvia Rubini,*[§] *Anna Fontcuberta i Morral,*[¶,#] *and Marta De Luca*[*,‡]

[†]Physics Department, University of Basel, Basel, 4056, Switzerland

[‡]Physics Department, Sapienza Università di Roma, Rome, 00185, Italy

[¶]Laboratory of Semiconductor Materials, Institute of Materials, EPFL, Lausanne, 1015, Switzerland

[§]CNR- Istituto Officina dei Materiali (IOM), Laboratorio TASC, Trieste, 34149, Italy

[∥]Physics Department, University of Trieste, Trieste, 34127, Italy

[⊥]Interdisciplinary Center for Electron Microscopy, EPFL, Lausanne, 1015, Switzerland

[#]Faculty of Basic Sciences, Institute of Physics, EPFL, Lausanne, 1015, Switzerland

[*]**Corresponding Author**
e-mail: marta.deluca@uniroma1.it

I. STRUCTURAL CHARACTERIZATION

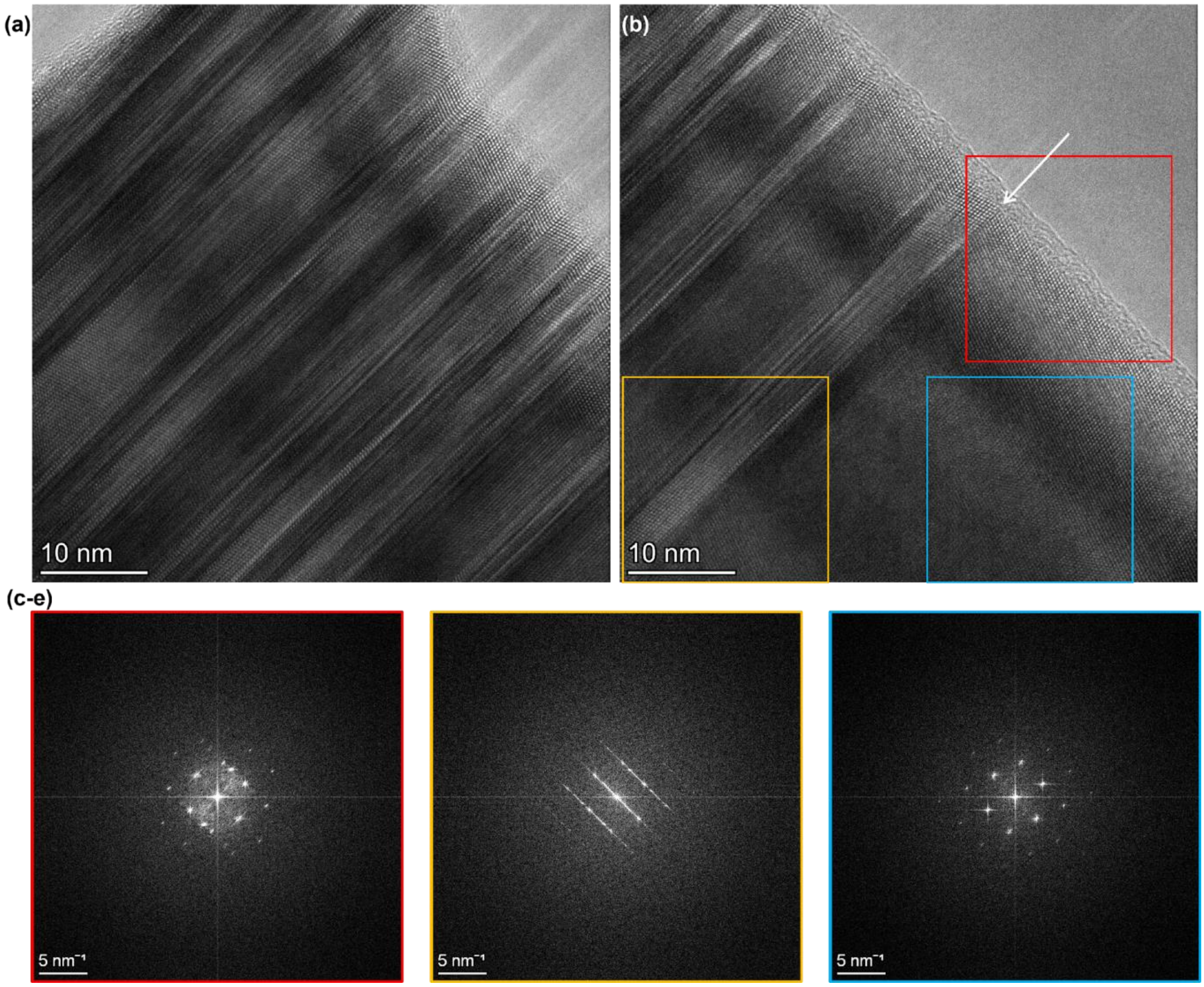

SI1. (a-b) show high resolution (HR)-TEM images taken from the bottom of a NW from sample A, in <110> zone axis. (c-e) show the Fourier transform images taken from regions illustrated with colored squares in (b). The bottom of the NW is very defective. Double diffraction spots in (c) are a signature for twin planes and streaks in (d) are stacking faults (SFs) below the start of the pure zincblende (ZB) phase of the middle region of the NW. The white arrow points to a twin plane in (b).

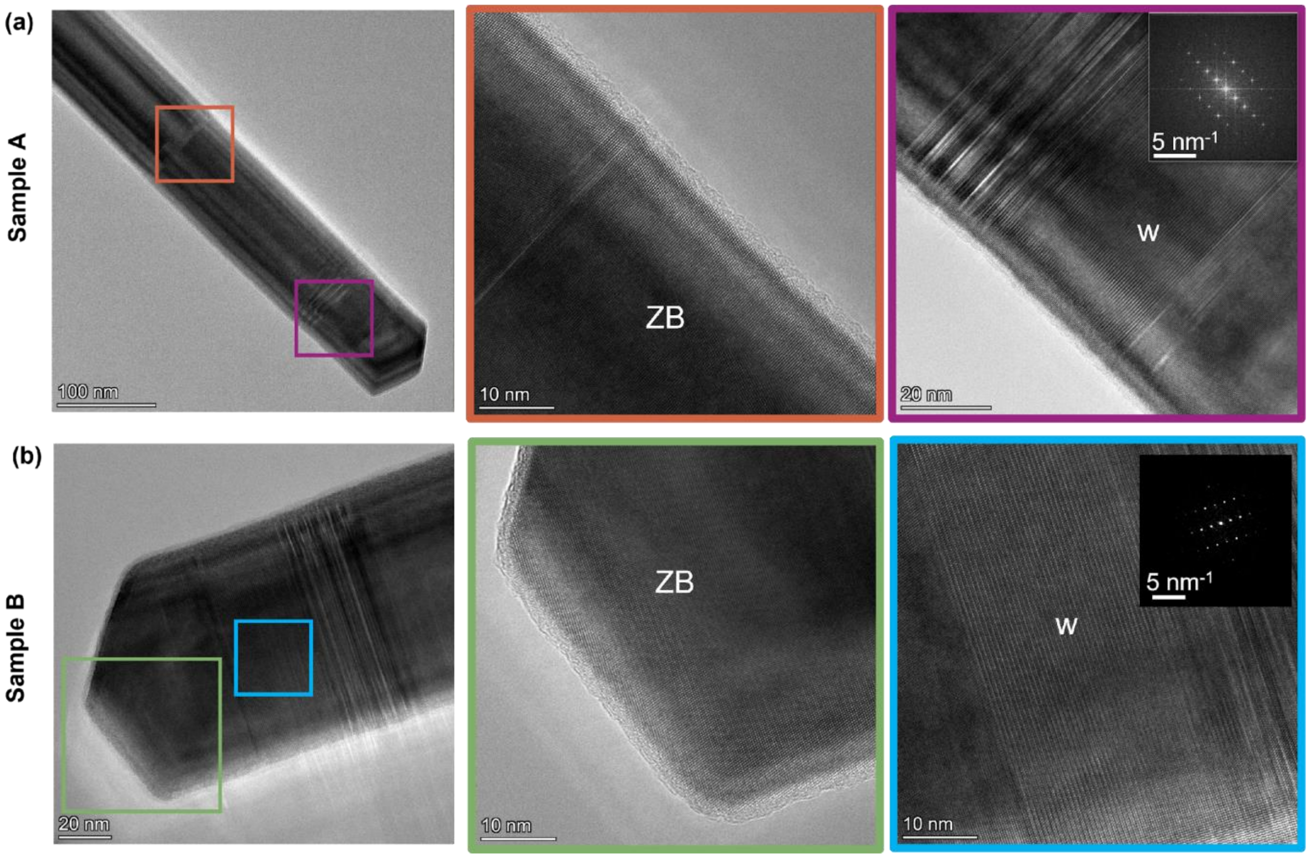

SI2. HR-TEM characterization of the tip regions of sample A (a) and sample B (b). SFs and wurtzite (WZ) sections are observed close to the tip, where WZ is indicated as 'W'. In self-catalyzed NWs, it is typical to observe these defects during the droplet consumption step [1,2].

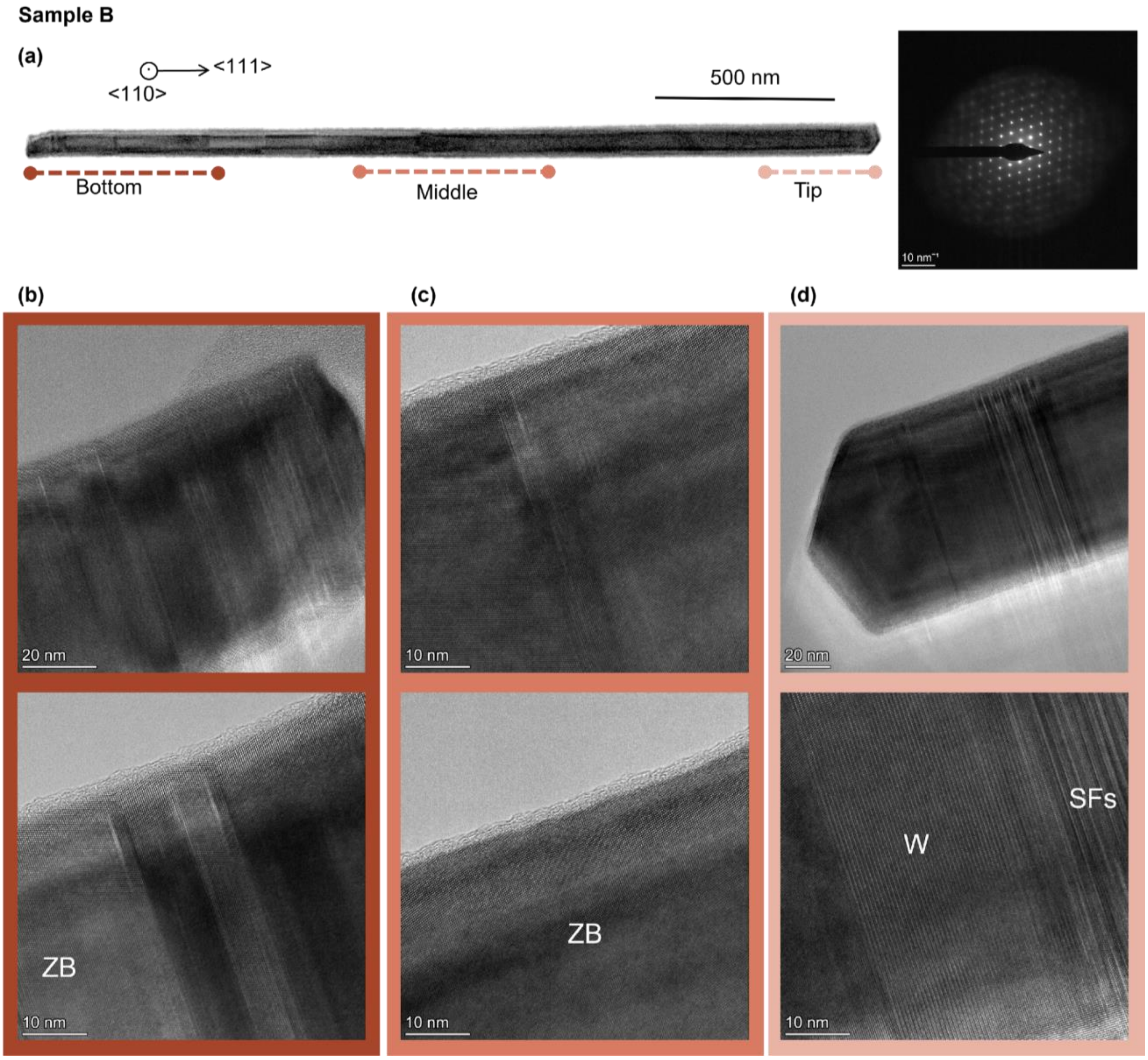

SI3. The bright-field TEM (BF-TEM) image of a NW from sample B and its selective area electron diffraction (SAED) pattern are given in (a). HR-TEM images are taken from the bottom (b), middle (c), and tip (d) sections. Top and bottom rows correspond to lower and higher magnification images, respectively. These images were taken from the <110> zone axis.

The detailed TEM characterization of NWs from sample B show similar structural characteristics as NWs from sample A. The bottom part is full of SFs, the middle region is pure and defect-free ZB crystal, and there is a WZ section at the tip.

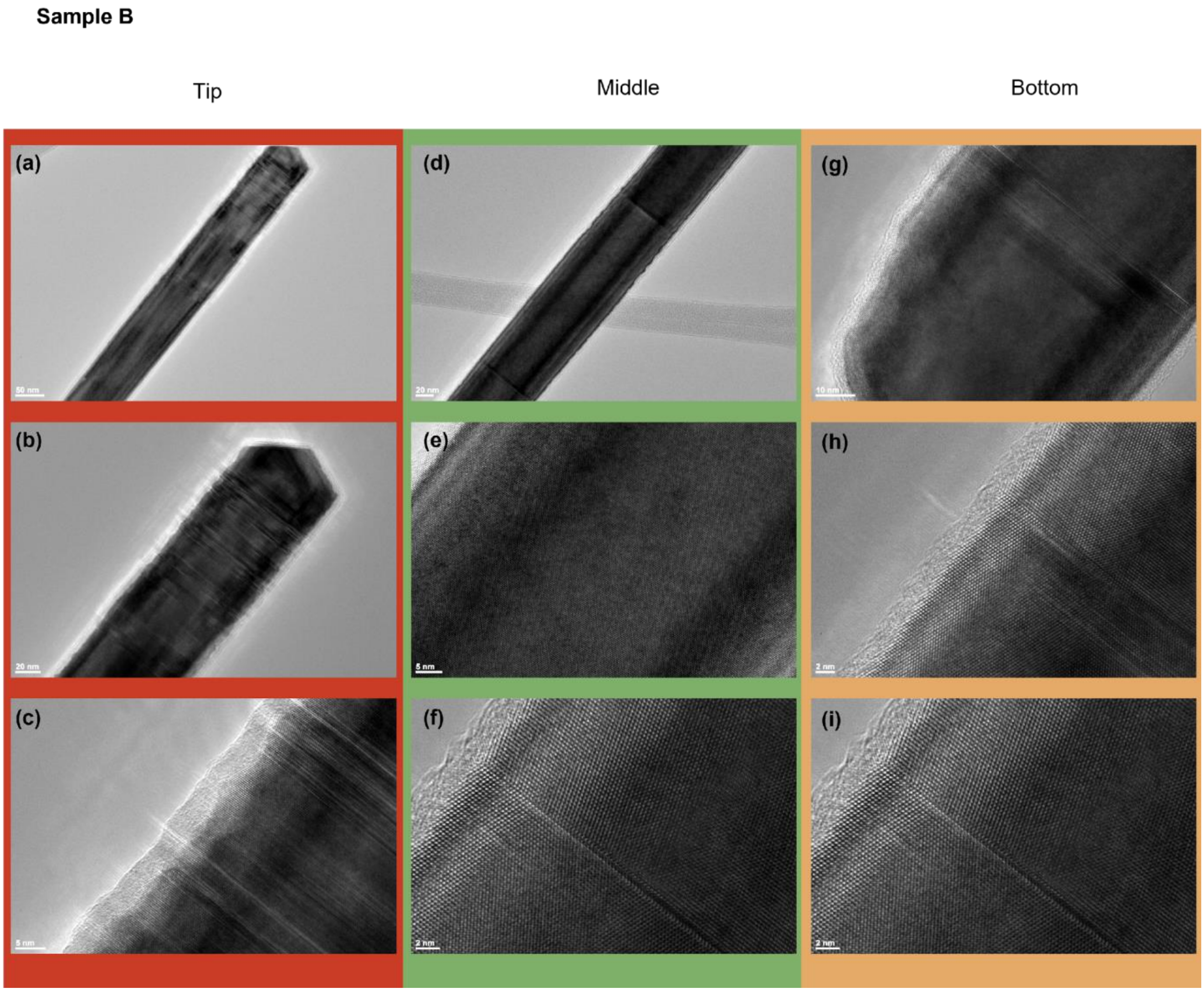

SI4. HR-TEM characterization of another wire from sample B for providing more statistics. Images taken from the tip regions are in panels (a)-(c), from the middle region in (d)-(f), and from the bottom region in (g)-(i). These images were taken from the <110> zone axis.

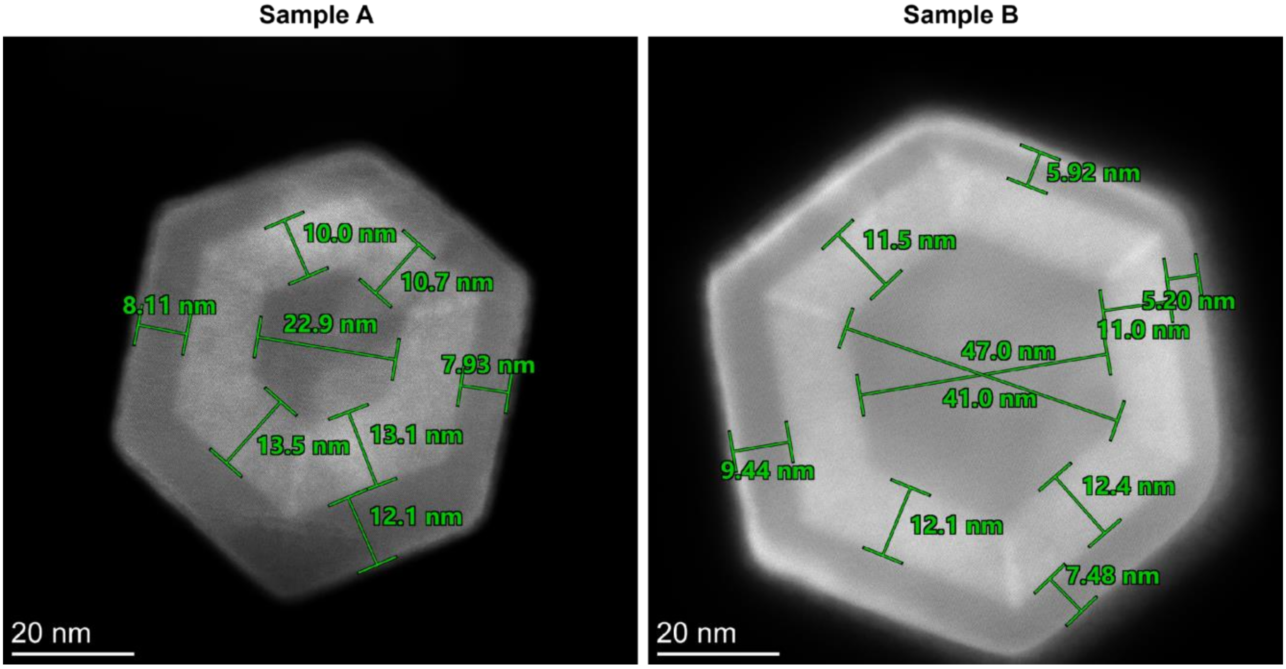

SI5. Annular dark-field (ADF)-STEM image of a cross-section from a NW of sample A in the left-side panel and sample B in the right-side panel. The measured widths are indicated for GaAs core, GaAsN shell, and GaAs outer-shell in several positions.

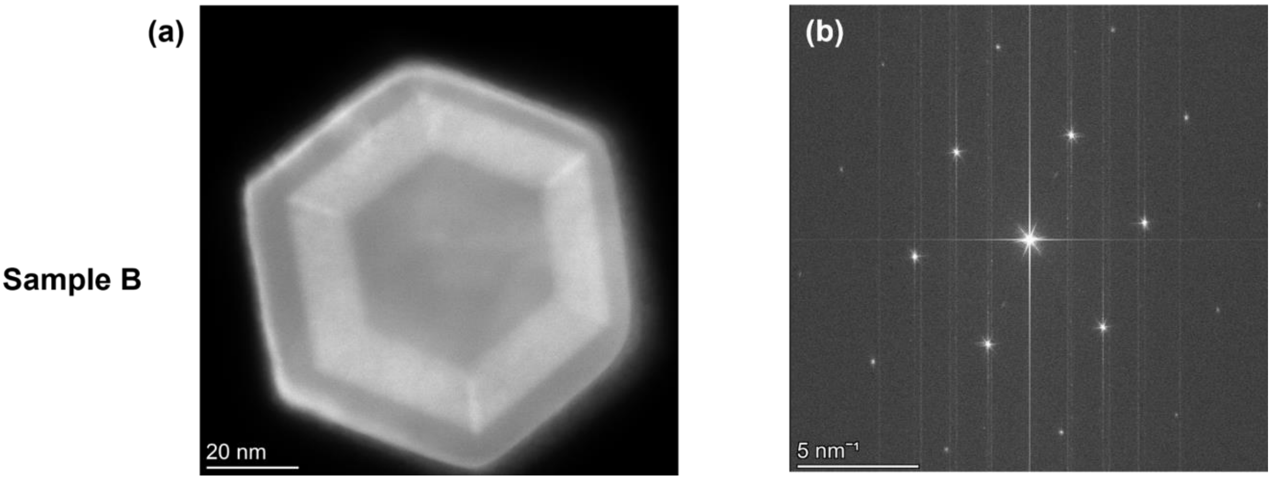

SI6. Cross-section image of sample B. (a) Atomic-resolution ADF-STEM image and (b) Fourier transform of the image, evidencing the ZB structure observed in <111> zone axis.

The presence of different contrasts along all six {112} planes laying in the symmetry axes linking the corners of the hexagonal shape of the NW has been reported before [3] for thicker core/shell NWs of the same kind, where authors attributed the presence of these lines to

diffraction contrasts and were not conclusive on the possible presence of small N concentration variation.

{112} planes are either A or B polar, meaning they are terminated by either Ga or As. Ternary alloys of III-V compounds show polarity-driven segregation of a preferential group III or group V element along specific polarity [4-7]. In these studies, the concentration of the third element in the alloy was high enough to be observed by the so-called mass-thickness contrast of HAADF-STEM imaging, that is sensitive to the atomic number (Z) of the material. In our case, no significant mass-thickness contrast variation is observed in HAADF conditions at the position of the three {112} planes of the GaAsN shell, showing a brighter contrast in the ADF-STEM image (see Figure SI6 a). It means a possible segregation of N in these planes is too small to be detected by HAADF imaging, as well as EDX (see Figure SI8), and only diffraction contrasts resulting from a subtle modification of the crystal lattice (e.g. from strain, defects, etc) are observed in the ADF image (Figure SI6 a) and SI7 b).

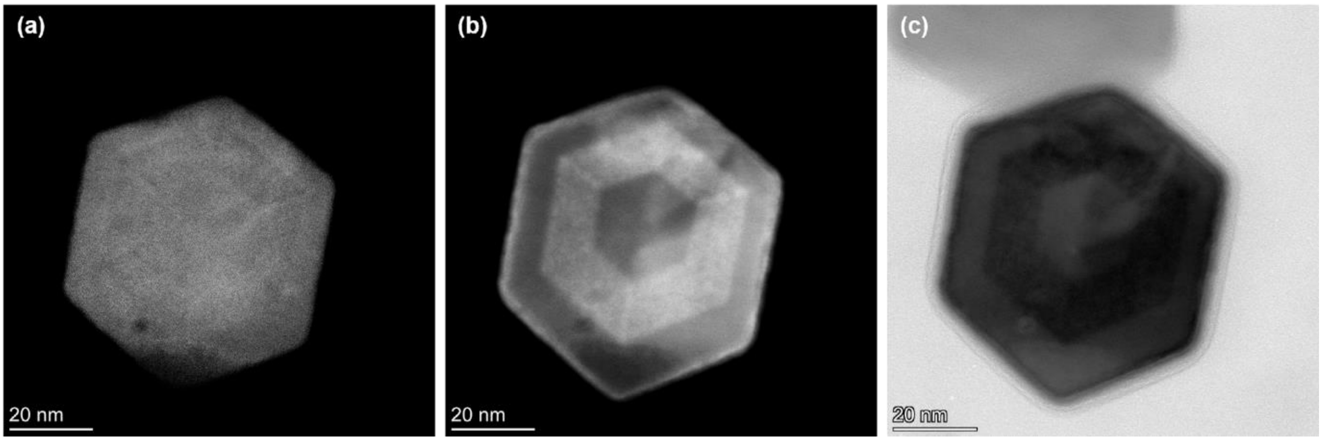

SI7. Supplementary cross-section images of a NW from sample A, using different STEM imaging conditions: (a) HAADF, (b) ADF, and (c) ABF.

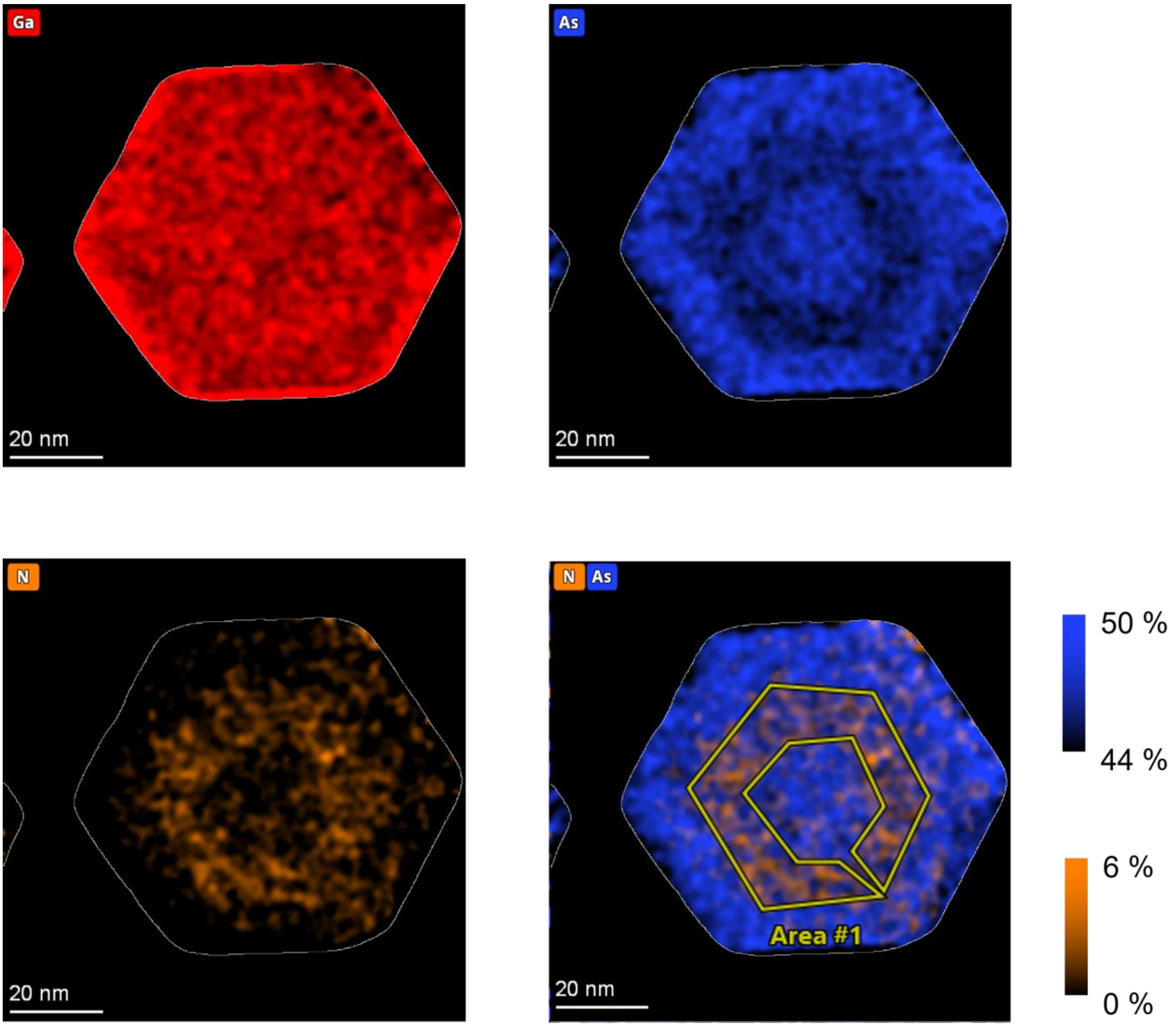

SI8. Quantitative energy dispersive x-ray spectroscopy (EDX) maps of a cross-section of the sample A, in atomic %. Red, blue, and orange correspond to Ga, As, and N elements, respectively. The elemental quantification from the EDX spectrum images was performed by Velox software, using the Cliff-Lorimer method. The Brown-Powell ionization cross-section model was used and absorption correction was taken into account, for a sample thickness of 97 nm. The sample thickness was measured by electron energy loss spectroscopy (EELS) from the inelastic/elastic scattering ratio, considering an inelastic mean free path of the 300 kV electrons of 138 nm [8]. Finally the mean atomic concentration in area #1, corresponding to the GaAsN shell, is 50.7, 46.4, and 2.9 atomic % for Ga, As, and N, respectively.

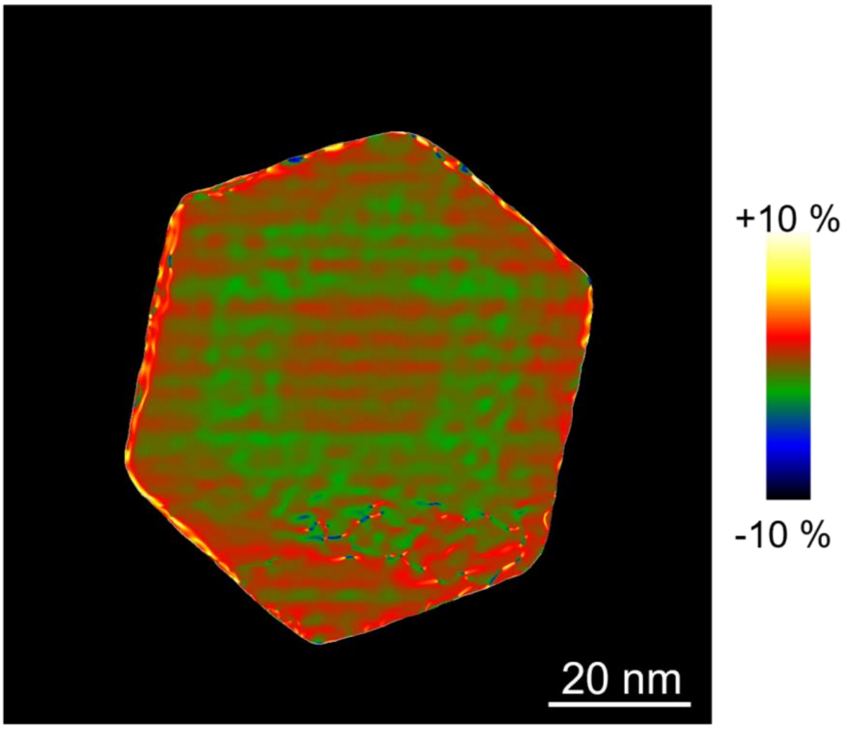

SI9. Strain analysis of sample A, by geometric phase analysis (GPA) of the atomic-resolution HAADF-STEM image [9]. Here the map of the mean dilatation of the crystal lattice is shown, calculated as $\frac{1}{2}(\varepsilon_{xx} + \varepsilon_{yy})$ with ε_{xx}, ε_{yy} being the strain in the x-, y-direction respectively. A negative dilatation of - 0.7% is observed in the GaAsN shell relative to the GaAs crystal. Using a refinement of the Vegard's law, this lattice contraction of 0.7% results from a N concentration of 2.5% in the GaAsN shell for sample A, which matches closely with our EDX data above. For more details, GPA analysis was performed using in-house scripts written for GMS 3 software. We used a lattice constant of GaAs (a_{GaAs}) of 5.6535 Å, and the deviation from the Vegard's law of GaAsN reported in reference [10] and expressed as: $a_{GaAs(1-x)Nx} = a_{GaAs} - 1.0605x - 20.95x^2$.

II. BAND ANTI-CROSSING MODEL AND BAND-STRUCTURE ALIGNMENT IN THE NANOWIRE HETEROSTRUCTURE

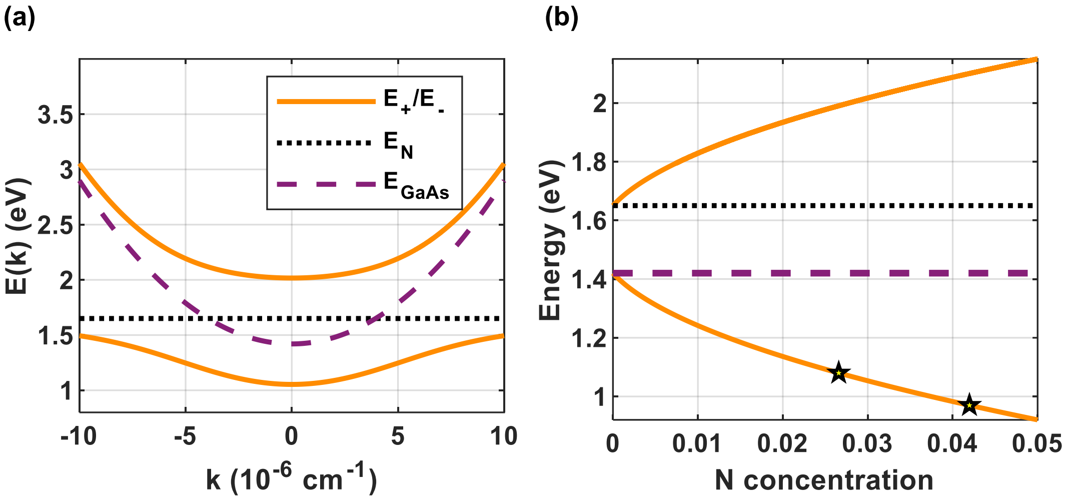

SI10. Band-anti-crossing model for dilute GaAsN. The bandgap energy of GaAsN decreases with increasing N concentration due to a perturbation of the conduction band states (orange lines in panels a and b) by localized N states (black dashed line), whose energy is in resonance with the GaAs conduction band (purple dashed line). This leads to a splitting of the conduction band into an upper and lower band, and thereby to a reduction of the bandgap energy, as shown in (a). The dispersion relation is given by $E_{\pm}(k) = \frac{1}{2}(E^N + E^C(k) \pm \sqrt{(E^N - E^C(k)^2 + 4V^2 \cdot x)}$, where E^N and E^C are the energies of the N-states and of the conduction band minimum with respect to the valence band maximum. V is the interaction potential that accounts for the mixing of the two states, and x is the N concentration [11,12]. For increasing N concentration, the bandgap energy is reduced further, as shown by the orange line in (b), which shows the conduction band energy (at k=0) of the upper and lower bands, $E_{\pm}$, as a function of N concentration. The GaAsN emission band is centered around 1.015 eV in the NWs of this work, which would correspond to a N concentration of 3.4% (if any other effects, such as strain, quantum confinement in the quantum well, or possible changes of the valence band energy due to the presence of N are neglected). An energy range centered on the maximum of the photoluminescence (PL) emission intensity at room temperature (RT), and having width equal to the FWHM of the PL peak (shown in Figure 1 (c) of the main paper) is delimited by the stars displayed in panel (b), indicating N concentration fluctuations ranging approximately from 2.7 to 4.1%.

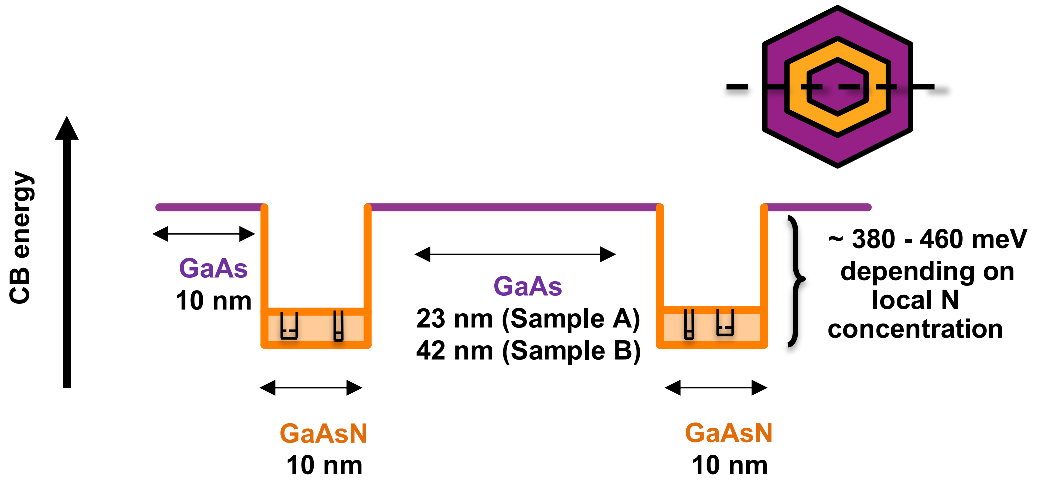

SI11. Illustration of the conduction band (CB) energy (with respect to the valence band maximum) over a line through the cross-section of the GaAs/GaAsN/GaAs core/shell/shell NW heterostructure as indicated by the dashed line in the image of the top right. The GaAs (high bandgap material) is represented in purple, and the GaAsN (low bandgap material) is represented in orange. A blueshift of the PL energy due to confinement is expected for the GaAsN quantum well, as discussed around Figure 1 of the main paper and not shown in the illustration. No radial confinement in the higher bandgap GaAs core is expected for NWs with a total diameter of approximately 60 nm [13]. The exact reduction of the bandgap energy in the GaAsN material depends on the local N concentration, as shown in Fig. SI10; the indicated 380 - 460 meV reduction of the GaAs bandgap corresponds to the difference between the GaAs regions and the GaAsN shell emission for the N% estimated in Fig. 1(c) of the main paper and in SI10. The energy range is shaded in orange and determined by the FWHM of the GaAsN emission band, which is caused by local N concentration fluctuations within the GaAsN shell (for simplicity, the interplay with possible size fluctuations of the quantum well are not considered here). These local concentration fluctuations result in three-dimensional confinement and in the creation of quantum dot-like states within the GaAsN shell, as illustrated by the potential wells drawn in black.

III. ADDITIONAL PL MEASUREMENTS OF SAMPLES A and B

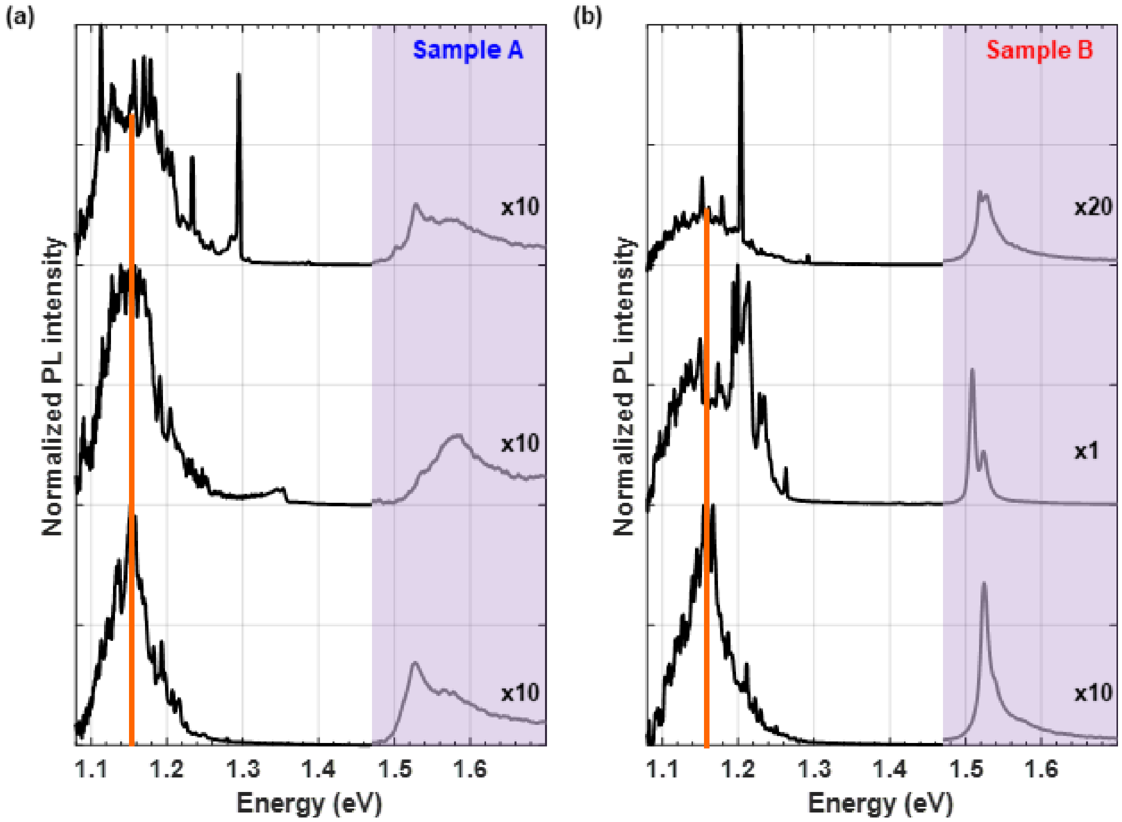

SI12. μ-PL spectra taken at T=6K on typical points on the growth chip for sample A (with a thin core of 20 nm) in (a) and for sample B (with a thicker core of 40 nm) in (b). The GaAs-core emission (see shadowed high-energy region beyond 1.48 eV) is multiplied by the indicated factor for better visibility. The orange line on the GaAsN emission band is a guide to the eye. These spectra were measured with the CCD detector; due to the low efficiency of the detector in the low-energy range, the intensity of the low-energy band of the GaAsN emission is partially cut, modifying the lineshape and introducing an upshift. The PL signal is collected from about 3-8 NWs in each point.

All the points show the two emission bands characteristic for these samples: One at low energy, emitted by the GaAsN shell and spiked with sharp peaks, and one band centered between 1.51-1.57 eV, emitted from the GaAs core. The GaAsN emission energy does not shift when moving the sub-micrometric laser spot on different points of the ensemble, which indicates a relatively uniform average N concentration (within the laser spot size) among different NWs. The broad GaAsN band measured at every point can arise from the local

inhomogeneity in N composition (within the FWHM discussed in SI10) or by other size-dependent fluctuations (*e.g.*, different shell thickness or different N% in the NW corners with respect to the NW side facets). Fig. SI12 also shows that the energy of the sharp lines varies when the signal is emitted from different points or from different NWs. This leads us to conclude that the sharp lines are not caused by N-complexes involving several atoms—emitting at several well-defined energies [14] —but rather by N-concentration fluctuations in the shell of each NW, leading to dips at different energies in the electronic potential and confining the carriers in three dimensions, as illustrated in SI11.

The PL emission of the GaAs core has been multiplied by the factors indicated in the figure to make the signal visible, as the GaAs-core emission is much weaker than the emission from the GaAsN shell, due to the fact that carriers recombine mostly in the low bandgap energy regions. The GaAs core emits at energies of 1.53 eV and 1.57 eV for sample A and at 1.51 eV and 1.53 eV for sample B. Two mechanisms can cause this blueshift with respect to the GaAs bulk emission of 1.515 eV. In thin GaAs NWs, charge carriers have been found to thermalize at temperatures much higher than the lattice temperature, which can thermally activate charge carriers to higher band states [15] and lead to a blueshift. Furthermore, the lattice mismatch between GaAs and GaAsN leads to a compressive strain in the GaAs, which is expected to cause a blueshift of the GaAs energy with a strong dependence on the core diameter. This strain distribution could be confirmed by Raman spectroscopy measurements as it was done in other core-shell NWs [16]; however, Raman measurements are hardly performed in our thin NWs. Both aforementioned mechanisms significantly impact a thinner GaAs core, which explains the more robust blueshift present in sample A and the double-band emission observed in the GaAs core signal from these NW samples. A dominant emission around 1.57eV has also been measured by cathodoluminescence on the polytypic tips of GaAs NWs [17].

IV. TEMPERATURE DEPENDENT PL MEASUREMENTS ON SOME ADDITIONAL QDs

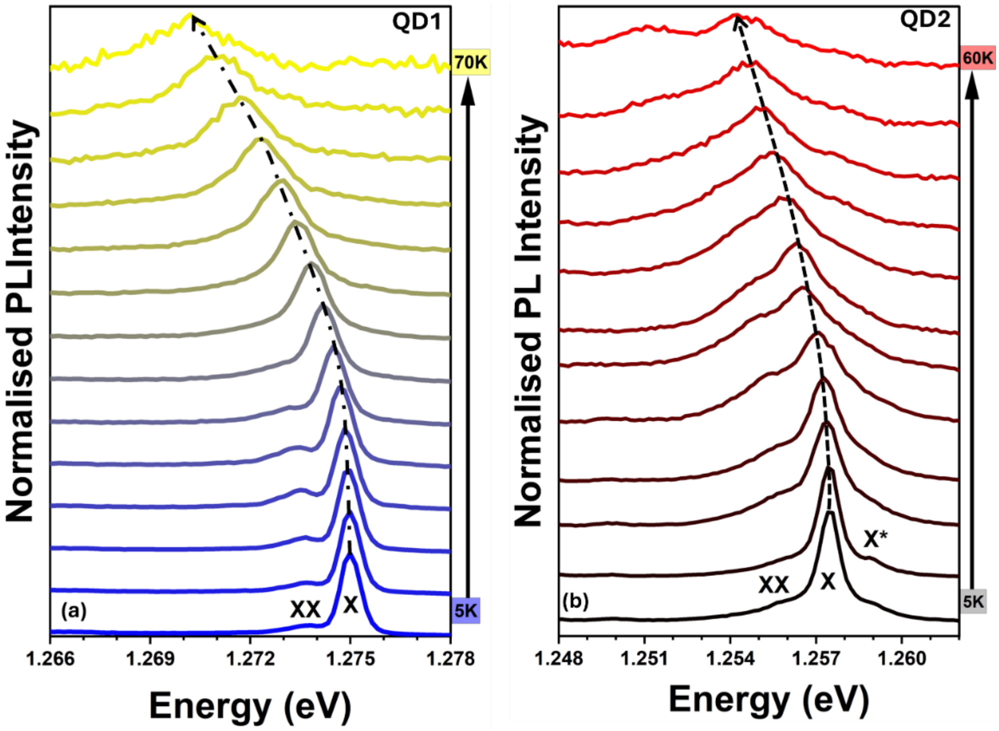

SI13. Temperature-dependent μ-PL measurements performed on two single, isolated quantum dot (QD) lines. These lines, having energies of 1.275 eV (QD1) and 1.257 eV (QD2), were acquired at two different points of sample B, with laser excitation power of 0.5 μW for QD1 and of 0.75 μW for QD2. The PL signal from both QDs is quenched at around 60-70K, similar to the quenching temperature of QD3, presented in the main text [see Fig. 4(c)]. At the lowest temperature, the luminescence from the QDs can be resolved into different features denoted as: X (exciton), XX (bi-exciton), and X* (charged exciton) recombination. The intensity of these excitonic transitions decreases with increasing temperature, accompanied by a linewidth broadening and by a peak-energy shift to the lower energy side. The peaks are well isolated and fitted with a Gaussian (QD1 and QD3) or Lorentz (QD2) type fitting function, depending on the emission lineshape of the QDs. A more detailed analysis of the temperature evolution of the different parameters characterizing these QD emissions is provided in SI14.

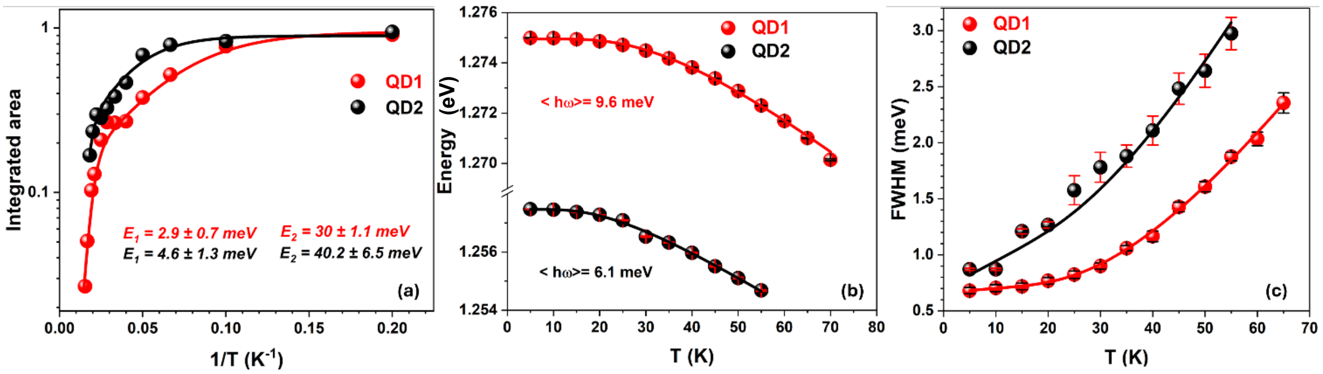

SI14. Analysis of the temperature-dependent PL of QD1 and QD2. (a) Arrhenius plot of integrated intensity of the X peak vs. 1/T, fitted with a two-activation-energy (E_1 and E_2) model. (b) Evolution of X emission energy with temperature, fitted with a one-oscillator model. (c) Change of the FWHM of the X emission with temperature, fitted with the exciton-phonon coupling model to extract the contribution of different phonons responsible for linewidth broadening. All fitting functions are described in the text below.

To study the carrier localization in the QD compared to the localized excitons in the GaAsN shell, the temperature study shown in Figures 4 (b) and (c) of the main paper is fitted using an Arrhenius equation with two activation energies, given by

$$I(T) = \frac{I_0}{1 + Ae^{-E_1/k_B T} + Be^{-E_2/k_B T}}$$

where I_0 is the intensity at 0 K, T is the temperature, E_1 and E_2 are activation energies and A and B their intensity contributions [18].

Figure SI14 shows further statistics for Arrhenius fits of different QD lines. We find values of E_1 between ~3-6 meV, which is close to the GaAs exciton binding energy [19], but it is unlikely that this value is related to the exciton ionization; it is more likely that it is ascribable to non radiative centers, usually related to defects. We find values for E_2 between ~30-40 meV, larger than what we found for the GaAsN band [see Fig. 4(d) of the main paper]. The extracted value of E_2 for all QDs suggests a stronger carrier confinement at the localized, isolated potential dips making up the spectrally isolated QDs with respect to the GaAsN shell region.

The redshift of the QD emission energy as a function of temperature, plotted in Fig. SI14 (b) and in Fig. 4 (c) of the main paper, is analysed using the one oscillator model [20], given as:

$$E(T) = E(T=0) - S < \hbar w > [\coth\left(\frac{< \hbar w >}{2K_B T}\right) - 1]$$

where S is a dimensionless coupling constant and <$\hbar w$> is the average phonon energy. This model provides a reasonable understanding of how phonons influence the temperature-dependent emission energy. The average phonon energy derived from this model for all the QDs ranges between 6 and 10 meV, indicating a similar interaction between excitons and phonons. This suggests that the primary phonon modes affecting the emission energy as temperature changes are consistent across these QDs, confirming a common origin for the QDs' formation.

The evolution of the FWHM of QD emission with temperature, plotted in Fig. SI14 (c) and in Fig. 4 (c) of the main paper, has been fitted using the exciton-phonon coupling model [21]:

$$\Gamma(T) = \Gamma_0 + y_a T + \frac{y_l}{exp^{(\frac{T_{LO}}{T})} - 1}$$

where $\Gamma(T)$ is the temperature-dependent FWHM and Γ_0 is the temperature independent FWHM (it depends on the fluctuations of the QD confinement potential). The y_a is described as a linear acoustic phonon coupling constant, y_l as an optical phonon coupling constant, and T_{LO} represents the average optical phonon temperature. The extracted values of these coupling constants range between 3.7 - 25 μeV/K for y_a and between 5.5 - 8 meV for y_l. The T_{LO} values range between 120 - 170K (10 - 14.5 meV). The values obtained for the phonon-coupling constants suggest that the exciton-optical phonon coupling provides the most significant contribution to the spectral linewidth broadening, as is typical of III-V crystals.

V. POWER DEPENDENT PL MEASUREMENTS ON SOME ADDITIONAL QDs

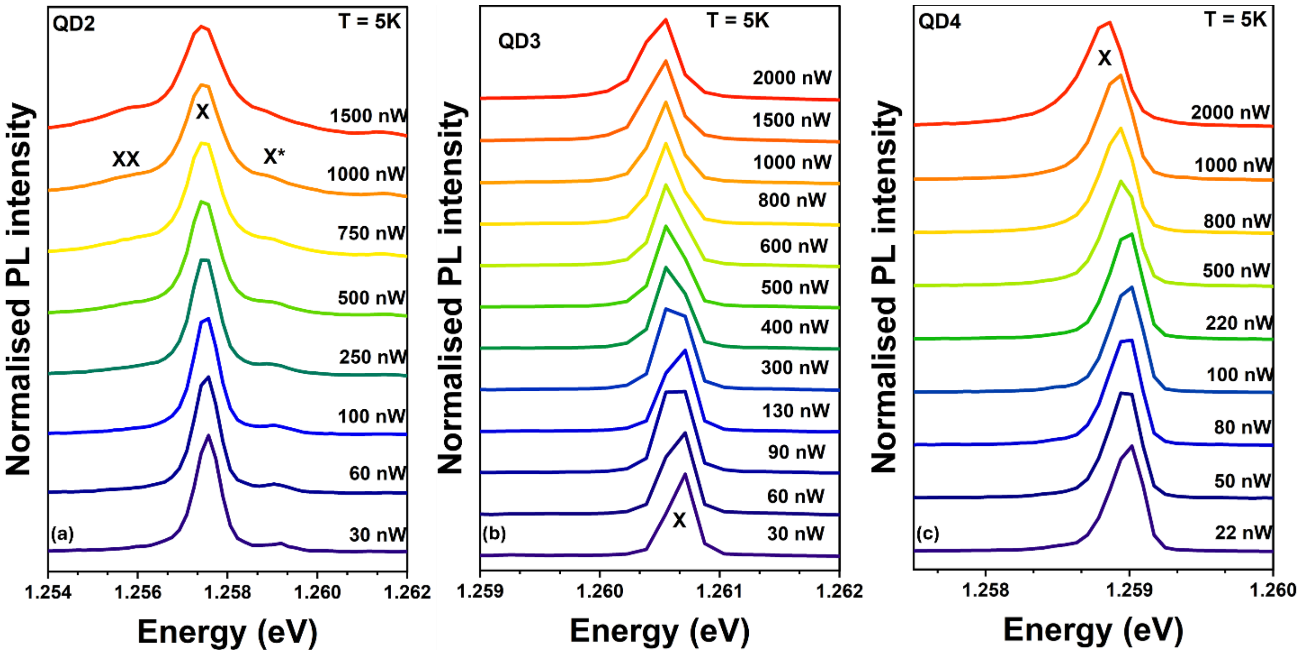

SI15. *T*=5K power-dependent μ-PL spectra of three different single isolated QDs: (a) QD2, (b) QD3, (c) QD4. Neutral excitons (X) dominate at low power and exhibit a small redshift and broadening for increasing power; in QD2 with increasing power also other excitonic recombination mechanisms participate in the luminescence, labelled based on the analysis summarized in Fig. SI17.

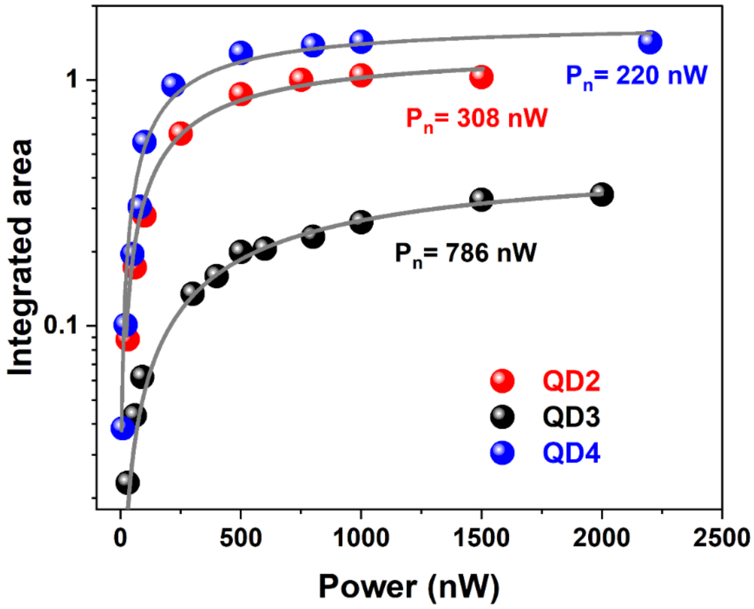

SI16. Semi-log plot of the integrated area of the X emission line of three QDs as a function of laser excitation power, showing saturation behaviour at high power, which is well reproduced by the fitting function $I = I_{sat}\,[P/(P+P_n)]$ (where I_{sat} is the saturation intensity, P is the laser power and P_n is the laser power for which the intensity is half of the saturation value). At the lowest power, the PL intensity increases linearly with excitation power, as shown in Fig. SI17. When the excitation power is increased, as more and more states within the QD become occupied, the rate of radiative recombination can decrease, leading to a saturation of the PL intensity. This phenomenon is described as a state-filling effect at high excitation power.

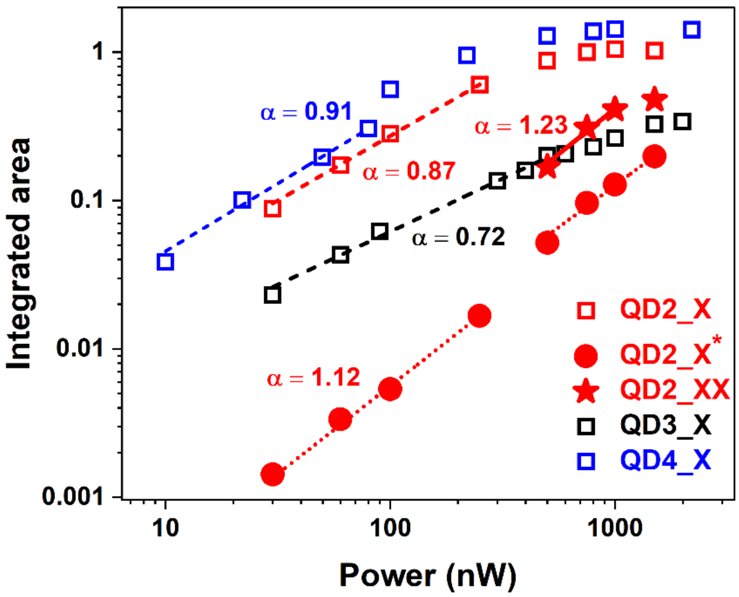

SI17. Log-log plot of the integrated area as a function of the excitation power for the individual X emission lines of the PL spectra shown in Fig. SI15. The data follow a power-law dependence expressed as $I \propto P^{\alpha}$, where I stands for the integrated PL intensity and P for the excitation power. The slope α of the fit indicates the possible origin of the different emission lines observed in the QD emission spectra. For all QDs, the slope of the dominant emission line ranges between 0.7-0.9, suggesting an excitonic nature of the emission line (labelled X). The line which has lower emission energy has α~1.2, almost double than the value of the excitonic emission, and is thus attributed to a bi-excitonic emission (XX). The α value for the other emission line is ~1.1, and it is thus tentatively attributed to a charged exciton emission (X*).

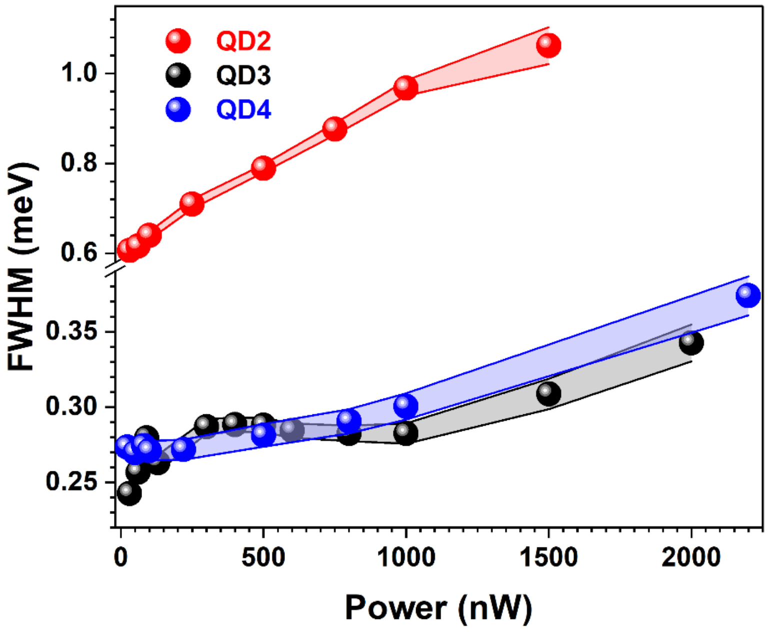

SI18. Evolution of the QD emission linewidth (FWHM) as a function of the excitation power for the X excitonic emission line of the three QDs presented in Figs. SI15-17. The FWHM ranges between 250 and 700 μeV for all the QDs. The linewidth is increasing with the increase in excitation power, usually due to excitation power-driven spectral diffusion and, to some extent, to local heating. The shadowed regions indicate the error bars.

The power-induced linewidth broadening in a QD can be due to random fluctuations of the local electric field near the dot surroundings [22], which implies a varying Stark shift of the energy levels of the confined excitons (light-driven spectral diffusion effect) [23]. The redshift seen in Fig. SI15 at high powers also suggests an increase of the average local field with increasing power. Also heating effects due to high power can lead to some redshift.

Concerning the low-power value of the QDs' FWHM, it is between 250 and 700 μeV for all QDs. This linewidth is not Fourier-limited, thus it is not related only to the emitter radiative lifetime. It depends also on other mechanisms, such as fluctuation of charges near the surface, which are the main broadening mechanisms in nanostructures like NWs. Increasing NW diameter could reduce this linewidth, both because it can lead to waveguiding, and because it would protect the QD from the surface effects.

VI. TIME-RESOLVED PL MEASUREMENTS ON SOME ADDITIONAL QDs

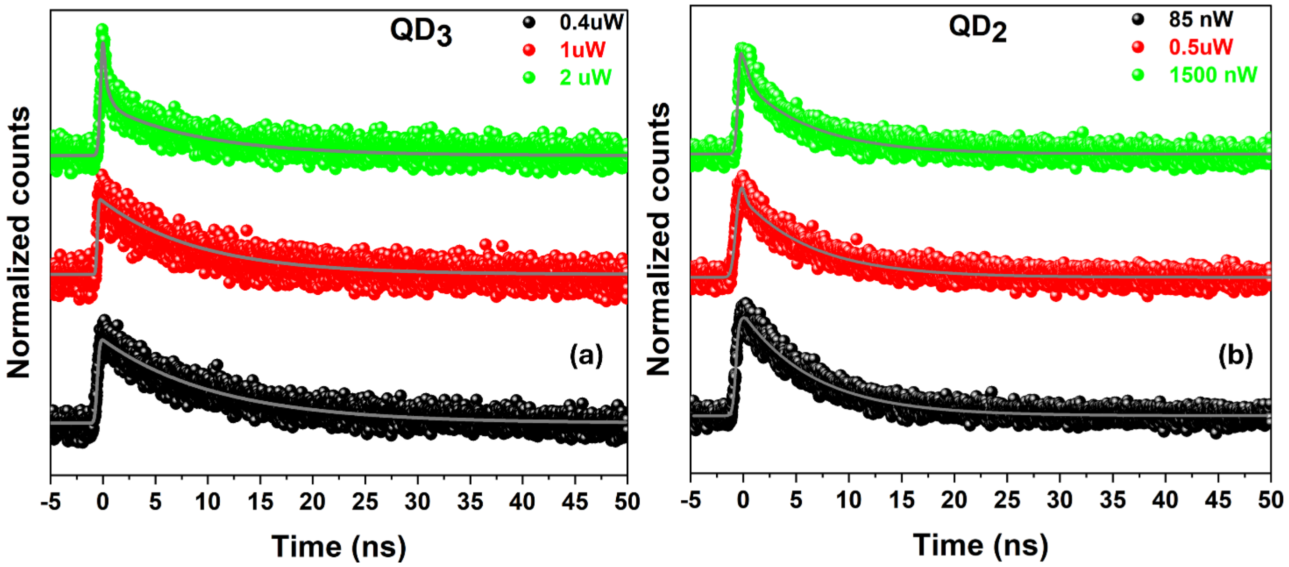

SI19. µ-TRPL transient decay of the dominant emission line X of two QDs (QD3 in panel (a) and QD2 in (b)), measured at 5K as a function of the excitation power density (from very low to the saturation power of the QD emission, see Fig. SI16). The standard bi-exponential model, resulting in the grey lines, was used to fit the TRPL decay to extract the excitonic decay component (slower decay), as shown in Fig. 5 (c) of the main paper.

To fit the time-resolved PL (TRPL) decay traces displayed in Fig. SI19 and in Fig. 5 (c) of the main paper, we have used the function below:

$$\mathbf{Y}\,(x) = y_0 + \left[\left(\frac{A}{\left(w * \sqrt{\frac{\pi}{2}}\right)}\right) * exp^{\left(-2*\left(\frac{(x-x_0)}{w}\right)^2\right)}\right] + \left[\left\{\left(A1 * exp^{\left(\frac{-x}{\tau_1}\right)}\right) + \left(A2 * exp^{\left(\frac{-x}{\tau_2}\right)}\right)\right\} * \left(1 + erf\left(\frac{(x - x_0)}{t_l}\right)\right)\right]$$

where x is time, y_0 the baseline, x_0 the time offset. The first term after the offset term is denoted as the Gaussian term, to consider the rise in the TRPL signal at zero time delay with amplitude *A*. The second term contains the fast (τ_1) and slow decay (τ_2) components with amplitudes *A1* and *A2* along with the error function, which is crucial for the model optimization process by minimising the value of the error function at time (t_l) and providing the best fit to the experimental data. The evolution of the extracted decay time with excitation power is discussed in the main text.

VII. CATHODOLUMINESCENCE MEASUREMENTS

(a) (b)

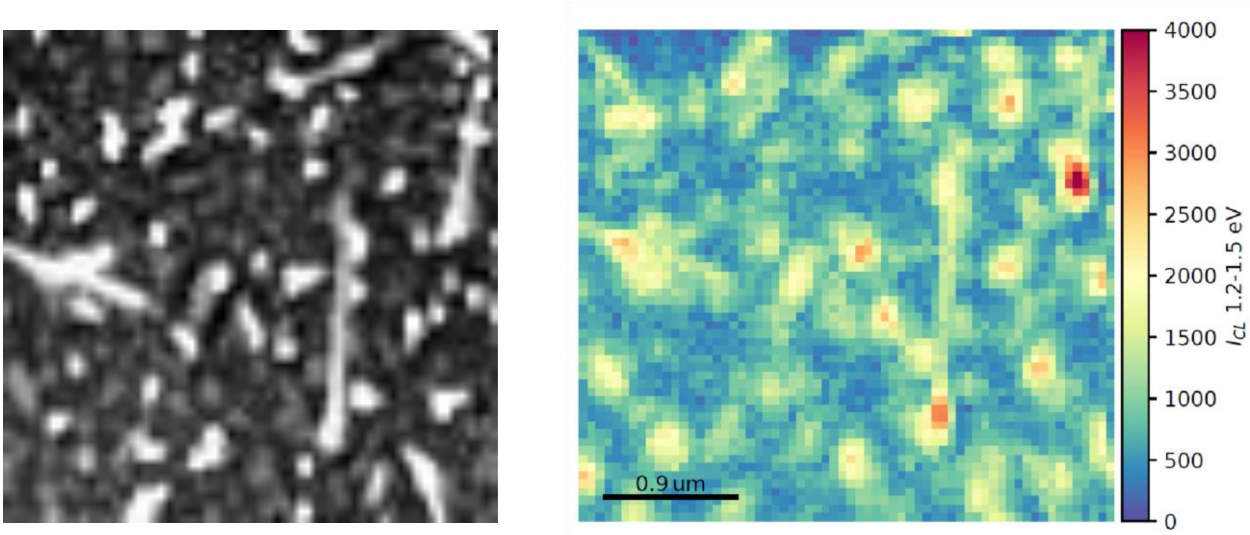

SI20. Cathodoluminescence measurements of sample B confirm that the emission from both the GaAsN shell and the GaAs core is from single vertical NWs connected to their growth substrate since there is a 1-to-1 correspondence between SEM (left side panel) and cathodoluminescence images (right side panel). In the cathodoluminescence map, the signal was integrated from 1.2 to 1.5 eV to show the spatial dependence of the overall optical emission.

Cathodoluminescence measurements shown in Figure SI20 allow ruling out that the QD signal investigated in PL arises from substrate. A further proof could be obtained by measuring single horizontal NWs. However, while the optical emission of vertical NWs connected to the growth chip was intense, the thin NWs from sample A and B lacked emission when being transferred onto a foreign substrate. This is linked to the strong heating that is typical of thin GaAs-like NWs [15] and to a lack of heat evacuation due to the small thermal conductivity and the large contact resistance of the nanosized NW with the Si substrate. In order to still measure spatially resolved μ-PL emission along a single transferred NW, which can be important to correlate optical and structural properties of the NWs, it will be important to increase the total NW diameter. This will require careful optimization of growth parameters to be able to keep the defect-free structure.

VIII. DATA ANALYSIS OF $g^2(\tau)$ MEASUREMENTS AND $g^2(\tau)$ FOR SOME ADDITIONAL QDs

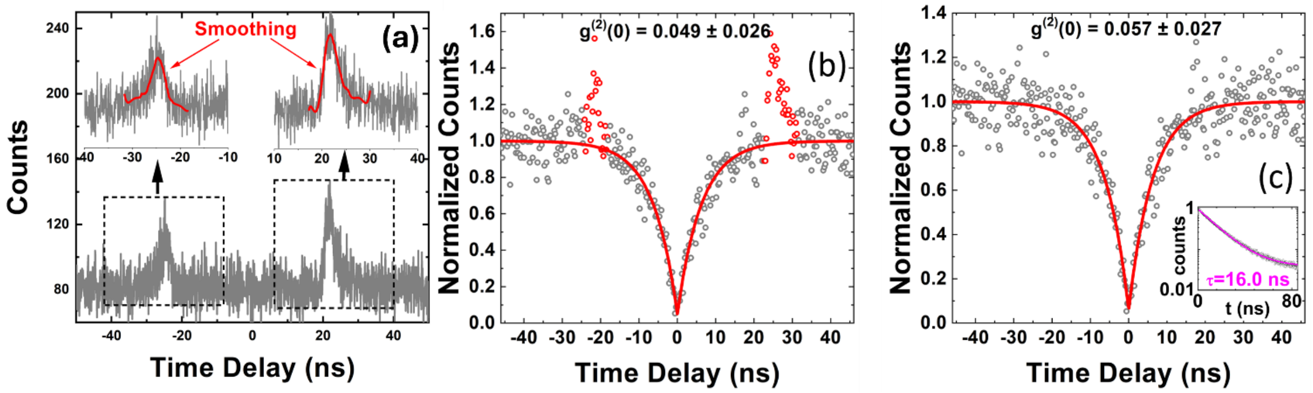

SI21. Crosstalk removal in the CW second-order autocorrelation function ($g^2(\tau)$) of the QD emission. (a) $g^2(\tau)$ of a bulk InP sample, which shows two crosstalk peaks on both sides of the zero time delay. The inset displays smoothing of the crosstalk peaks. (b) $g^2(\tau)$ of one of our QD emission lines (QD4) measured at T=5K, which has crosstalk peaks (red dots) at the same time delay as in InP. The $g^2(\tau)$ fit performed by just masking the crosstalk data gives the value of $g^2(0)$= 0.049±0.026. (c) After removing the crosstalk, we run again the fit and obtain $g^2(0)$= 0.057±0.027 for QD4. The inset shows a TRPL measurement of this QD line, from which a decay time of τ=16 ns is extracted.

In case of $g^2(\tau)$ measurements in an energy range close to the emission range of the material composing the detector, some photons emitted by the detector may reach the other detector and introduce false coincidences. It is possible to remove them to extract the true sample correlation function. We used the subtractive method to cancel this crosstalk. As shown in Fig. SI21 (a), the crosstalk is estimated by running a $g^2(\tau)$ measurement on a bulk material (here InP at room temperature) with the same alignment as the QD measurement. The $g^2(\tau)$ signal on InP does not show any antibunching dip at τ=0, because the sample is not a quantum light emitter, but it shows two side peaks on both sides of zero delay (which further proves that these peaks come from the setup). These peaks are first smoothed, then subtracted from the QD $g^2(\tau)$ signal, which thus will no longer show pronounced crosstalk peaks, see panel (c). This operation does not affect much the $g^2(0)$ value, as it can be seen by comparing panels (b) and (c).

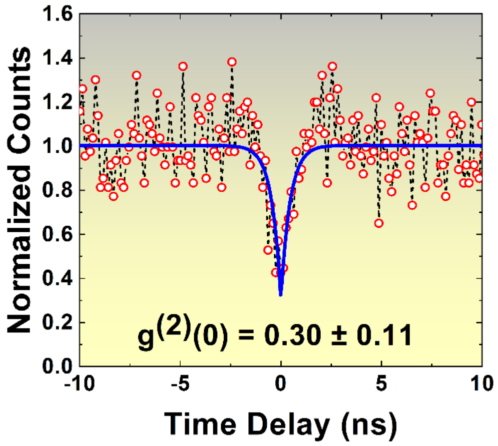

SI22. $g^2(\tau)$ data and fit of an additional QD emission line at 1.246 eV (QD5) performed in CW at *T*=5K. The normalized coincidence counts are shown as a function of the time delay. Single-photon emission is confirmed by a value of g^2 (0) equal to 0.30±0.11.

IX. FITTING OF $g^2(\tau)$ MEASUREMENTS USING RATE EQUATIONS

To fit the CW $g^2(\tau)$ measurement in Figure 5 (e) in the main paper and to model the pulsed $g^2(\tau)$ measurement in Figure 5 (f) we have considered that the time evolution of the population of carriers inside our QDs can be obtained by solving the following system of rate equations:

$$\frac{dp_{00}}{dt} = -2.\frac{p_{00}}{\tau_c} + \frac{p_{11}}{\tau_d}$$

$$\frac{dp_{10}}{dt} = \frac{(p_{00} - p_{10})}{\tau_c}$$

$$\frac{dp_{01}}{dt} = \frac{(p_{00} - p_{01})}{\tau_c}$$

$$\frac{dp_{11}}{dt} = \frac{(p_{01} + p_{10})}{\tau_c} - p_{11}\left(\frac{1}{\tau_d} + \frac{1}{\tau_{MS}}\right) + \frac{p_{MS}}{\tau_{MS}}$$

$$\frac{dp_{MS}}{dt} = (p_{11} - p_{MS})\,\frac{1}{\tau_{MS}}$$

where $p_{\{ij\}}$ is the probability of finding the system in a state with *i* electrons and *j* holes, τ_c is the carrier capture time for the QD (assumed to be the same for electrons and holes), and τ_d is the decay time for the exciton. For the sake of simplicity, *i* and *j* were both fixed at 1, which is equivalent to the assumption that our QD is populated, at most, by a single electron-hole pair, *i.e.*, by a single exciton. Although in contradiction with the fact that even the simplest QD system —with a single confined state for both electrons and holes— should always have the ability to host at least two electrons and two holes (that is, a biexciton), this simplification is consistent with the PL spectrum of our QDs. At the excitation power levels employed for autocorrelation measurements, indeed, such a spectrum is still unequivocally dominated by the single X peak, which is much more intense than the XX or X* features.
As discussed in refs. [24,25] for $\tau \geq 0$ ($\tau \leq 0$) the CW exciton autocorrelation, $g^2(\tau)$, is equal to $p_{11}(\tau)$ [$p_{11}(-\tau)$], obtained by solving the system of rate equations shown above, with the initial condition that $p_{00}(\tau=0) = 1$ (*i.e.*, at $\tau=0$, immediately after the recombination of an exciton, the QD must be in its empty state). Experimentally, it is interesting to note that the $g^2(\tau)$ in Fig. 5 (e) in the main paper is characterized by the presence of broad bunching peaks on both sides of the antibunching dip at $\tau=0$. To reproduce this feature, an additional metastable state had

to be introduced, exchanging excitons with the QD with a characteristic time τ_{MS}. By "metastable", we mean that the decay time of this extra-state is much larger than τ_{MS} (so much larger that such decay time could be set to infinity in the equations).

After convolution with the instrumental response that we measured, the g²(τ) curve obtained with the procedure discussed above was fitted to the experimental data, using τ_c and τ_{MS} as free parameters. τ_d was kept fixed to 5.25 ns, the value obtained from fitting the time-resolved curve acquired with an excitation power of 0.6 μW (for time-resolved and CW/pulsed excitation autocorrelation measurements, power levels were adjusted by looking at the saturation level of the X peak, see Fig. 5 (b)). The choice of fixing τ_d was dictated both by the need to minimise the number of fitting parameters and by the desire to obtain a set of values that would univocally describe the time evolution of the QD emission, across all available experimental datasets. The curve resulting from this fit, displayed in Fig. 5 (e) of the main text, corresponds to τ_c = 2.22 ns and to τ_{MS} = 68.5 ns.

For pulsed excitation, on the other hand, the exciton autocorrelation function can be obtained by solving a system of rate equations nearly identical to that displayed above (only the excitation mechanism changes slightly to account for the presence of a pulsed laser), following the procedure detailed in [25]. The g²(τ) curve displayed in Fig. 5 (f) of the main text is generated through this procedure, using the values of τ_d, τ_c, and τ_{MS} obtained from fitting the time-resolved and CW autocorrelation data. The good overlap of this curve (which, we must reiterate, is not a fit, as there are no free parameters involved) with the experimental data further validates our analysis.

X. ESTIMATION OF QD EFFICIENCY

For all our single QD lines, the luminescence intensities detected at 5K lie in the range 500-5000 counts per second on the CCD detector, for an excitation power below saturation (which ranges between 0.5-1 μW, depending on the specific QD).

To estimate the intrinsic photon extraction efficiency of the QDs, it is necessary to measure the setup efficiency (η_{setup}) at the QD wavelength. We used a pulsed laser with the same wavelength and a similar bandwidth as our QD and measured the effective transmission of each optical component in the setup, resulting in η_{setup} = 7%. The QD extraction efficiency, $\eta_{extrac.}$, is given by [26]

$$\eta_{extrac.} = \frac{QD\ counts/s\ on\ the\ APD\ detectors}{\eta_{det} * \eta_{setup} * R_{laser}}$$

where η_{det} is the APD detector efficiency at the QD emission wavelength (in our case equal to 18%) and R_{laser} is the repetition rate of the laser (using a pulsed laser with repetition rate 39 MHz and wavelength 525 nm). The estimated efficiency is equal to 1.02%. This quite low value is however very promising, because these thin NWs do not support waveguiding effects. Typically, NWs whose diameter is ~ 0.22 x the wavelength of light can support guided modes. To obtain waveguiding in our NWs, we should grow thicker NW diameters (~200 nm). Alternatively, we could use external cavities—and, for those, it would be useful to control the energy of the QDs, which is typically possible in GaAsN materials by post-growth hydrogenation [25].

XI. GROWTH CONDITIONS

Sample	GaAs core Ga flux, ML/s	GaAs core As/Ga beam equivalent pressure ratio	GaAs core growth time	Shell Ga flux, ML/s	Shell As/Ga beam equivalent pressure ratio	Total pressure GaAsN shell (mTorr)	Plasma source power (W)	GaAsN shell growth time	GaAs shell growth time
A	0.2	19.5	20’	1	8.3	$5*10^{-5}$	80	4’30”	4’30”
B	0.2	19.5	20’	1	11.1	$3.6*10^{-5}$	60	4’30”	4’ 30”

SI23. Growth parameters for GaAs/GaAsN NW samples.

With the given growth parameters, the nominal N content estimated in pseudomorphic thin films on (001) GaAs grown in same conditions as the NWs would be 1.5% for sample A and 0.9% for sample B. However, the N incorporation is expected to differ with respect to the pseudomorphic thin films due to different growth dynamics in this thin NW geometry and due to the NW sidewalls being <110> directed [27], therefore we estimated the effective N content in each sample by PL measurements. Our results suggest a higher capability of NWs to incorporate N with respect to thin films, to be further investigated.

REFERENCES

[1] G. Priante, S. Ambrosini, V. G. Dubrovskii, A. Franciosi, and S. Rubini, Crystal Growth & Design 13, 3976 (2013).
[2] S. Ambrosini, M. Fanetti, V. Grillo, A. Franciosi, and S. Rubini, AIP Advances 1, 042142 (2011)
[3] Y. Araki, M. Yamaguchi, and F. Ishikawa, Nanotechnology 24, 065601 (2013).
[4] R. Bergamaschini, F. Montalenti, and L. Miglio, Applied Surface Science 517, 146056 (2020).
[5] C. Zheng, J. Wong-Leung, Q. Gao, H. H. Tan, C. Jagadish, and J. Etheridge, Nano Letters 13, 3742 (2013).
[6] Y. Zhang, A. M. Sanchez, J. Wu, M. Aagesen, J. V. Holm, R. Beanland, T. Ward, and H. Liu, Nano Letters 15, 3128
(2015).
[7] L. Francaviglia, G. Tütüncüoglu, S. Martí-Sánchez, E. Di Russo, S. Escobar Steinvall, J. Segura Ruiz, H. Potts, M. Friedl, L. Rigutti, J. Arbiol, and A. Fontcuberta I Morral, Physical Review Materials 3, 023001 (2019).
[8] R. Egerton, *Electron Energy-Loss Spectroscopy in the Electron Microscope* (Springer US, Boston, MA, 2011).
[9] Hÿtch, M. J., Snoeck, E. & Kilaas, R. Quantitative measurement of displacement and strain fields from HREM micrographs. Ultramicroscopy 74, 131 (1998).
[10] W. Li, M. Pessa, and J. Likonen, Appl. Phys. Lett. 78, 2864 (2001).
[11] W. Shan, W. Walukiewicz, J. W. Ager, E. E. Haller, J. F. Geisz, D. J. Friedman, J. M. Olson, S. R. Kurtz, Phys. Rev. Lett. 82, 1221 (1999).
[12] Vurgaftman, J. R. Meyer, L. R. Ram-Mohan, J. Appl. Phys. 89, 5815 (2001).
[13] B. Loitsch, D. Rudolph, S. Morkötter, M. Döblinger, G. Grimaldi, L. Hanschke, S. Matich, E. Parzinger, U. Wurstbauer, G. Abstreiter, J. J. Finley, G. Koblmüller, Adv. Mater. 27, 2195 (2015).
[14] X. Liu, M. E. Pistol, and L. Samuelson, Phys. Rev. B 42, 7504 (1990).
[15] D. Tedeschi, M. De Luca, H. A. Fonseka, Q. Gao, F. Mura, H. H. Tan, S. Rubini, F. Martelli, C. Jagadish, M. Capizzi, and A. Polimeni, Nano Lett. 16, 3085 (2016).
[16] L. Balaghi, G. Bussone, R. Grifone, R. Hübner, J. Grenzer, M. Ghorbani-Asl, A. V. Krasheninnikov, H. Schneider, M. Helm, and E. Dimakis, Nat. Comm. 10, 2793 (2019).
[17] C. Somaschini, S. Bietti, A. Trampert, U. Jahn, C. Hauswald, H. Riechert, S. Sanguinetti, and L. Geelhaar, Nano Lett. 13, 3607 (2013).
[18] D. Bimberg, M. Sondergeld, E. Grobe, Phys. Rev. B, 4 (10), 3451 (1971).
[19] S. B. Nam, D. C. Reynolds, C. W. Litton, R. J. Almassy, T. C. Collins, C. M. Wolfe, Phys. Rev. B, 13 (2), 761 (1976).
[20] K. P. O'donnell, X. Chen, Appl. Phys. Lett., 58 (25), 2924 (1991).
[21] H. Zhao, S. Wachter, H. Kalt, Phys. Rev. B , 66 (8), 085337(2002).
[22] H. D. Robinson, B. B. Goldberg, Phys. Rev. B , 61 (8), R5086 (2000).
[23] S. A. Empedocles, D. J. Norris, M. G. Bawendi, Phys. Rev. Lett. 77 (18), 3873(1996).
[24] M. H. Baier, A. Malko, E. Pelucchi, D. Y.Oberli, E. Kapon, Phys. Rev. B 73 (20), 205321, (2006).

[25] M. Felici, G. Pettinari, F. Biccari, A. Boschetti, S. Younis, S. Birindelli, et al., Phys. Rev. B 101 (20), 205403 (2020).
[26] J. Neuwirth, F. Basso Basset, M. B. Rota, J.-G. Hartel, M. Sartison, et al.,Phys. Rev. B 106, L241402 (2022).
[27] J. Li, X. Han, C. Dong, C. Fan, Y. Ohshita, M. Yamaguchi, Journal of Alloys and Compounds 687, 42 (2016).
